# Schiff Base in Ketoamine Form and Rh(η^4^-cod)-Schiff Base Complex with Z′ = 2 Structure from Pairwise C-H···Metallochelate-π Contacts

**DOI:** 10.3390/molecules28010172

**Published:** 2022-12-25

**Authors:** Mohammed Enamullah, Imdadul Haque, Amina Khan Resma, Dennis Woschko, Christoph Janiak

**Affiliations:** 1 Department of Chemistry, Jahangirnagar University, Dhaka 1342, Bangladesh; 2 Institut für Anorganische Chemie und Strukturchemie, Universität Düsseldorf, Universitätsstr. 1, D-40225 Düsseldorf, Germany

**Keywords:** Rh(η^4^-cod)-Schiff base complexes, Z′ = 2 structure, keto-enol tautomerism, thermal analysis, DFT/TD-DFT calculations

## Abstract

Condensation of 2-hydroxybenzaldehyde (salicylaldehyde) or 2-hydroxy-1-naphthaldehyde with 2-ethylaniline yields the Schiff base compound of (*E*)-2-(((2-ethylphenyl)imino)methyl)phenol (HL^1^) or (*E*)-1-(((2-ethylphenyl)imino)methyl)naphthalen-2-ol (HL^2^), which in turn react with the dinuclear complex of [Rh(η^4^-cod)(µ-O_2_CCH_3_)]_2_ (cod = cycloocta-1,5-diene) to afford the mononuclear (η^4^-cod){(*E*)-2-(((2-ethylphenyl)imino)methyl)phenolato-κ^2^N,O}rhodium(I), [Rh(η^4^-cod)(L^1^)] (**1**) or (η^4^-cod){(*E*)-1-(((2-ethylphenyl)imino)methyl)naphthalen-2-olato-κ^2^N,O}rhodium(I), [Rh(η^4^-cod)(L^2^)] (**2**) (L^1^ or L^2^ = deprotonated Schiff base ligand). The X-ray structure determination revealed that the HL^2^ exists in the solid state not as the usual (imine)N···H-O(phenol) form (enolamine form) but as the zwitterionic (imine)N-H^+^···^–^O(phenol) form (ketoamine form). ^1^H NMR spectra for HL^2^ in different solvents demonstrated the existence of keto-enol tautomerism (i.e., keto ⇆ enol equilibrium) in solution. The structure for **1** and **2** showed that the deprotonated Schiff base ligand coordinates to the Rh(η^4^-cod)-fragment as a six-membered N^O-chelate around the rhodium atom with a close-to-square-planar geometry. Two symmetry-independent molecules (with Rh1 and Rh2) were found in the asymmetric unit in **1** in a structure with Z’ = 2. The supramolecular packing in HL^2^ was organized by π-π and C-H···π contacts, while only two recognized C-H···π contacts were revealed in **1** and **2**. Remarkably, there were reciprocal or pairwise C-H···π contacts between a pair of each of the symmetry-independent molecules in **1**. This pairwise C-H contact to the Rh-N^O chelate (metalloaromatic) ring may be a reason for the two symmetry-independent molecules in **1**. Differential scanning calorimetry (DSC) analyses revealed an irreversible phase transformation from the crystalline-solid to the isotropic-liquid phase and subsequently confirmed the thermal stability of the compounds. Absorption spectra in solution were explained by excited state properties from DFT/TD-DFT calculations.

## 1. Introduction

The bidentate N,O-chelate (HL) or tetradentate N_2_,O_2_-chelate (H_2_L′) Schiff base ligands react with the dinuclear [Rh(η^4^-cod)(µ-X)]_2_ (cod = 1,5-cyclooctadiene; X = Cl, OMe, O_2_CMe) to give the mononuclear [Rh(η^4^-cod)(L)] or dinuclear [{Rh(η^4^-cod)}_2_(L′)] complexes [1,2,3,4,5,6,7,8,9,10]. These types of Rh(η^4^-cod)-Schiff base complexes have been used with excellent conversion and chemoselectivity towards hydroformylation and polymerization reactions [5,6,7,8,9,10]. Similar reactions with the chiral bidentate N,N-chelate Schiff base ligands generated the analogous chiral Rh(η^4^-cod)-Schiff bases complexes, which were used for the enantioselective hydrogenation or hydrosilylation of ketone derivatives [11,12,13,14,15,16]. Most of these studies evaluated catalytic activities of Rh(η^4^-cod)-complexes, which are significantly influenced by the structures of the catalyst as well as the coordination geometry around the rhodium atom [7,10,11,12,13,14]. Although the first article on Rh(η^4^-cod)-Schiff base complexes was published by Cozens et al. in 1971 [1], there was no structural information available until the molecular structure of dinuclear [{Rh(η^4^-cod)}_2_(L′)] {H_2_L′ = H_2_salophen = N,N′-(1,2-phenylene)bis(salicylideneimine)} was reported by Bonnaire et al. in 1982 [4]. The salophen ligand acts as a bridge between two Rh(I) atoms with a nearly square-planar geometry, giving a twisted conformation.

Our studies on Rh(η^4^-cod)-complexes with chiral N,O-chelate Schiff base ligands, synthesized from dinuclear [Rh(η^4^-cod)(µ-O_2_CMe)]_2_, showed considerable interest in their syntheses, molecular structures and catalytic properties [17,18,19,20,21]. Indeed, we first reported structurally elucidated chiral mononuclear [Rh(η^4^-cod)(L)] (L = salicylaldiminato/naphthaldiminato) [17,18]. Similar reaction with achiral N,O-chelate Schiff bases (HL) or with the tetradentate N_2_,O_2_-chelate Schiff bases (H_2_L′ = H_2_salen or H_2_salophen) gave the mononuclear [Rh(η^4^-cod)(L)] [19,20] or dinuclear [{Rh(η^4^-cod)}_2_(L′)] [20]. The X-ray molecular structure determination showed a six-membered N,O-chelation of the salicylaldiminates (including salen or salophen) or naphthaldiminates to the Rh(η^4^-cod)-fragment with distorted square planar geometry at the rhodium atom. Some of these complexes were tested for reduction of acetophenone into (*rac*)-1-phenyl-ethanol in the presence of diphenylsilane with conversions up to ca. 95% [18,21].

Extended studies on Rh(η^4^-cod)-complexes using chiral-amino acids/-amino alcohols instead of Schiff bases showed the interesting features of coordination and supramolecular chemistry [20,22,23]. In this connection, we have reported the mononuclear neutral [Rh(η^4^-cod)(AA)] (AA = chiral/achiral-amino carboxylato) and the cationic [Rh(η^4^-cod)(AOH)](O_2_CMe) (AOH = chiral-amino alcohol) [20,21,22]. The X-ray molecular structures showed a five-membered N,O-chelation of amino-carboxylate or amino-alcohol to the Rh(η^4^-cod)-fragment in distorted square planar geometry at the rhodium atom. Structural analyses further explored the observation that the achiral *N*-phenylglycinate provided a *racemate* of Rh(η^4^-cod)(*N*-phenylglycinate), with the nitrogen atom becoming the stereogenic center upon metal coordination. A two-fold spontaneous resolution of the *racemate* and its supramolecular packing provided two homo-chiral supramolecular helix-enantiomers, namely a (left-handed) *P*4_3_- and (right-handed) *P*4_1_-helical chain [20,22]. 

**Scheme 1:**
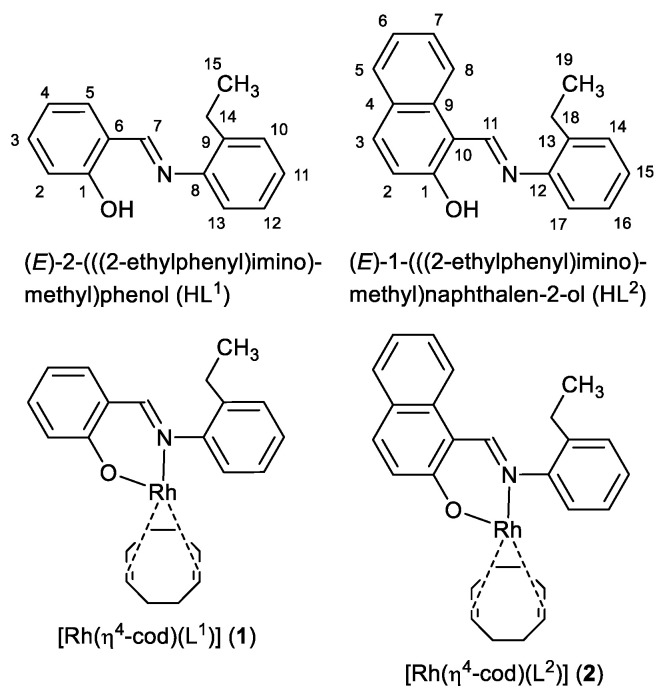
Formula of HL^1^, HL^2^ (with NMR atom numbering), **1** and **2**.

The present paper, in continuation, reports the results of synthesis, spectroscopy and molecular structures of the N,O-chelate Schiff bases (HL^1^ or HL^2^) and their complexes of [Rh(η^4^-cod)(L^1^)] (**1**) or [Rh(η^4^-cod)(L^2^)] (**2**) (Scheme 1). The molecular structures for HL^2^, **1** and **2** were elucidated by single-crystal X-ray diffraction and are discussed along with the supramolecular packing analysis. ^1^H NMR studies revealed HL^2^ to exhibit keto-enol tautomerism in solution, while at solid-state, it remains as the zwitterionic (imine)N-H^+^···^–^O(phenol) (ketoamine form). The optimized geometry and excited state properties were studied by DFT/TD-DFT and compared with the experimental results. 

## 2. Results and Discussion

**Scheme 2:**
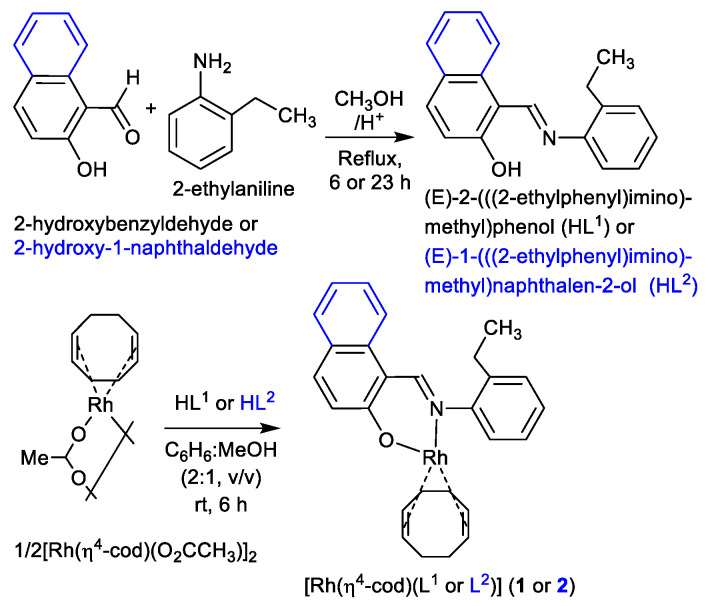
Synthetic route to the formation of the Schiff bases HL^1^ and HL^2^ as well as Rh(η^4^-cod)-Schiff bases complexes **1** and **2**.

The reaction of 2-hydroxybenzaldehyde (salicylaldehyde) or 2-hydroxy-1-naphthaldehyde with 2-ethylaniline gives the Schiff bases of (*E*)-2-(((2-ethylphenyl)imino)methyl)phenol (HL^1^) or (*E*)-1-(((2-ethylphenyl)imino)methyl)naphthalen-2-ol (HL^2^) (Scheme 2). These Schiff bases react with dinuclear (η^4^-cycloocta-1,5-diene)(acetato)rhodium(I), [Rh(η^4^-cod)(µ-O_2_CCH_3_)]_2_ in a mixture of C_6_H_6_:MeOH (2:1, *v*/*v*) to provide the mononuclear complexes of (η^4^-cod){(*E*)-2-(((2-ethylphenyl)imino)methyl)phenolato-κ^2^N,O}rhodium(I), [Rh(η^4^-cod)(L^1^)] (**1**) and (η^4^-cod){(*E*)-1-(((2-ethylphenyl)imino)methyl)naphthalen-2-olato-κ^2^N,O}rhodium(I), [Rh(η^4^-cod)(L^2^)] (**2**) (L^1^ or L^2^ = deprotonated Schiff base ligand) (Scheme 2). IR spectra showed the main characteristic imine band (νC=N) at 1616/1595 cm^−1^ (HL^1^) and 1609/1597 cm^−1^ (HL^2^) for the Schiff bases, which shift to 1609/1586 cm^−1^ (**1**) and 1616/1603 cm^−1^ (**2**) upon coordination to the metal ion. Electron impact (EI) mass spectra (Figure S1, Supplementary Materials) contained the molecular ion peak at m/z = 435 (**1**) and 485 (**2**) followed by several ion peaks for the Schiff base ligands and their fragmented species (see experimental section).

**Figure 1:**
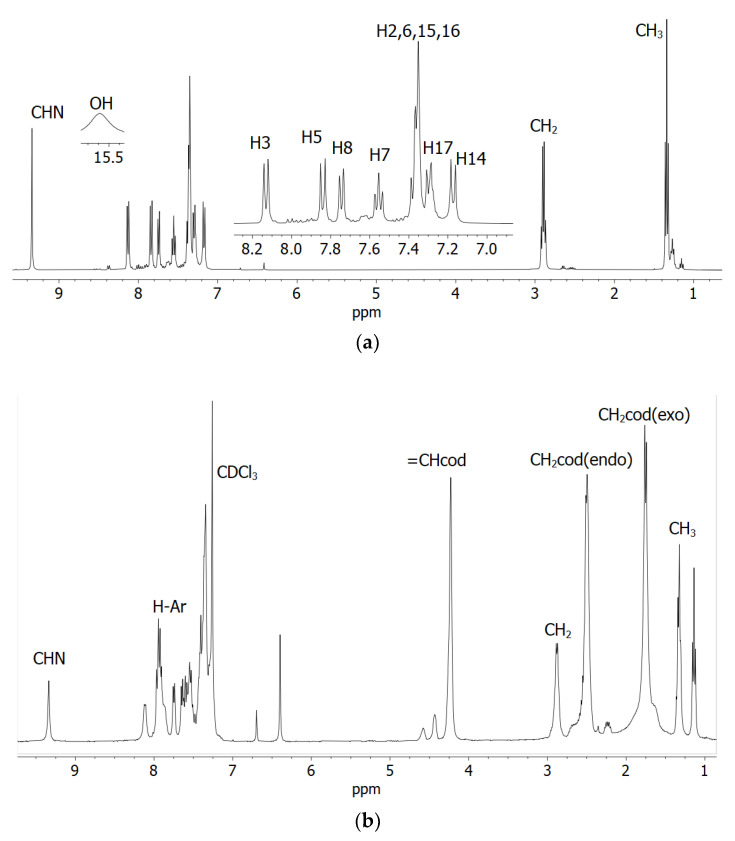
^1^H NMR spectra of the Schiff base (**a**) HL^2^ with hydrogen atoms numbered and (**b**) compound **2** in CDCl_3_ at 20 °C.

^1^H NMR spectra of the Schiff base ligands (HL^1^ and HL^2^) and the complexes (**1** and **2**) in CDCl_3_ are shown in Figure 1 (Figure S2 and Table 1), and they corresponded well to those of related Schiff bases and their Rh(η^4^-cod)-complexes [17,18,19,20,21]. Methyl (CH_3_) and methylene (CH_2_) protons appeared as a triplet at 1.24–1.34 ppm (J_HH_ = 7.6 Hz) and a quartet at 2.81–2.90 ppm (J_HH_ = 7.6 Hz) in the Schiff bases, respectively. These peaks were found as a triplet at 1.33 (**1**)/1.35 (**2**) ppm (J_HH_ = 7.6 Hz) and a quartet at 2.93 (**1**)/2.92 (**2**) ppm (J_HH_ = 6.8 Hz) in the complexes. The imine proton (CH=N) showed a singlet at 8.62 (HL^1^) and 9.34 (HL^2^) ppm for the Schiff bases and at 8.77 (**1**) and 9.36 (**2**) ppm for the complexes. The Schiff bases showed a broad peak at most downfield at 13.47 (HL^1^) and 15.63 ppm (HL^2^) for the phenolic proton (OH), which disappeared upon coordination to the metal ion. The broad peak corresponded to an exchange of the phenolic proton between the oxygen (enol-form) and nitrogen (keto-form) atoms, which resulted in a dynamic keto ⇆ enol tautomerism (i.e., keto ⇆ enol equilibrium) in solution as discussed below [24]. The presence of a naphthyl-ring in HL^2^ or **2** shifted the imine proton signal downfield by ca. 0.70 ppm in contrast to that in HL^1^ or **1** due to an electron donating inductive effect. Similarly, the phenolic proton signal shifted downfield by ca. 2.10 ppm in HL^2^ in contrast to that in HL^1^. ^1^H NMR spectra for the complexes showed signals for the exo- and endo-methylene protons (CH_2_cod_exo_ and CH_2_cod_endo_) of the rhodium-coordinated 1,5-cyclooctadiene as a multiplet at 1.77–1.79 and 2.52–2.54 ppm, respectively [17,18,19,20,21]. The four olefinic (CHcod) protons showed a single peak at 4.27 (**1**) and 4.26 (**2**) ppm, suggesting their symmetric nature in the coordination sphere [20,22,23,24]. In contrast, the analogous Rh(η^4^-cod)-salicylaldiminates/naphthaldiminates showed two or four peaks for four olefinic protons, indicating their asymmetric nature in the coordination sphere [17,18,19,20,21].

### 2.1. UV-Vis Spectra and Excited State Properties

**Figure 2:**
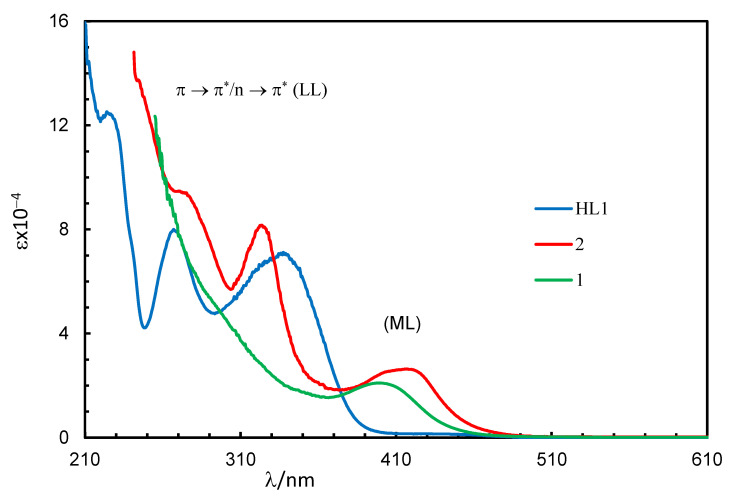
UV-Vis spectra for HL^1^ (0.10 mM), **1** (0.20 mM) and **2** (0.08 mM) in chloroform at 25 °C.

Absorption spectra for the Schiff base (HL^1^) and complexes (**1** and **2**) in chloroform are shown in Figure 2. The spectra for the complexes were almost identical, while they were a bit different from that of the Schiff base. The Schiff base showed three very strong bands below 400 nm with absorption maxima (λ_max_) at 227 nm (ε_max_ = 12,440 L mol^−1^ cm^−1^), 267 nm (ε_max_ = 8000 L mol^−1^ cm^−1^) and 341 nm (ε_max_ = 7030 L mol^−1^ cm^−1^) for different intra-ligand π→π^∗^/n→π^∗^ transitions (LL) [17,18,19,20,21]. The complexes showed these intra-ligand transitions bands below 365 nm with absorption maxima at λ_max_ = 323 nm (ε_max_ = 8163 L mol^−1^ cm^−1^) and 275 nm (9413 L mol^−1^ cm^−1^) for **2**, which were seen as a shoulder at ca. 310 nm for **1** due to shifting to the higher energy. The complexes further revealed a medium broad band at 365–500 nm with λ_max_ = 399 nm (ε_max_ = 2099 L mol^−1^ cm^−1^) for **1** and 417 nm (ε_max_ = 2638 L mol^−1^ cm^−1^) for **2.** This was due to metal–ligand charge transfer (ML) transitions based on the formation of [Rh(η^4^-cod)]^+^ and [Rh(L^1^ or L^2^)] species in [Rh(η^4^-cod)(L^1^ or L^2^)] (**1** or **2**), as reported for the analogous Rh(η^4^-cod)-Schiff bases/amino-acids complexes [17,18,19,20,21]. 

**Figure 3:**
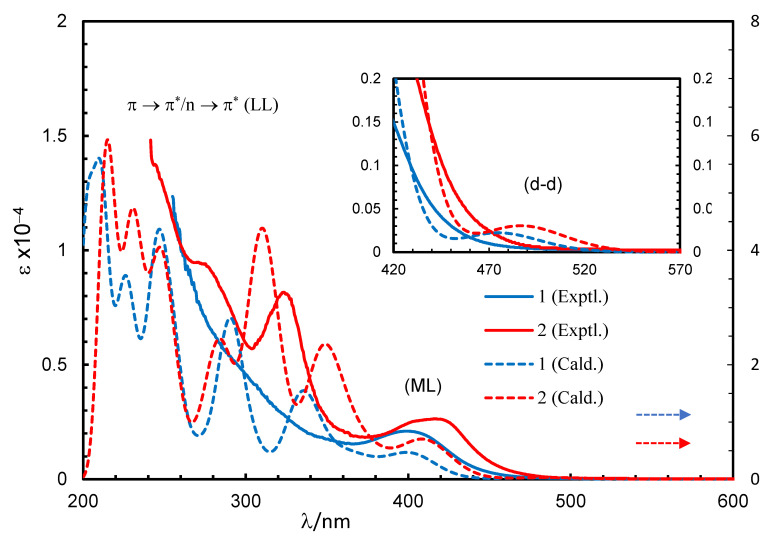
Experimental and simulated spectra for compounds **1** (0.20 mM) and **2** (0.08 mM) in chloroform (spectra simulated at B3LYP/SDD with PCM in chloroform). Gaussian band shape with exponential half-width s = 0.16 eV.

The excited state properties (simulated UV-Vis spectra) for compound **1** or **2**, calculated by TD-DFT at B3LYP/SDD, and the experimental spectra are shown in Figure 3. Some selected excited state properties and simplified assignments associated with the experimental bands are listed in Table 2 and discussed herein. The results showed that the excitation occurred from the combination of several MM, ML and LL transitions at a particular wavelength (excited state) (Tables S2 and S3). Indeed, excitation energies related to these transitions are very close, overlap with each other and make them difficult to interpret independently and in a simple manner [19,23,25,26]. Hence, a combined band consisting of MM (d-d) and ML transitions appeared at 475 (**1**) or 487 (**2**) nm, with the highest molecular orbital (MO) contribution of 98% (**1**) or 97% (**2**) and an oscillator strength (*f*) of 0.0022 or 0.0030 for the HOMO to LUMO transitions, respectively, (Figure 3, inset), which were not seen in the experimental spectra. Similarly, a combined band comprising all three transitions (MM, ML and LL) was found at λ_max_ = 402 (**1**) or 410 (**2**) nm, with the second highest MO contribution of 71% (**1**) or 76% (**2**) (*f* = 0.0426 or 0.0662) for the HOMO-1 to LUMO transitions, respectively, which were very close to the observed band at λ_max_ = 399 (**1**) or 417 (**2**) nm in the experimental spectra. In fact, the simulated spectra further indicated several very strong bands at shorter wavelengths with significant MO contributions and oscillator strength in parallel with the experimental spectra (Figure 3 and Table 2).

**Figure 4:**
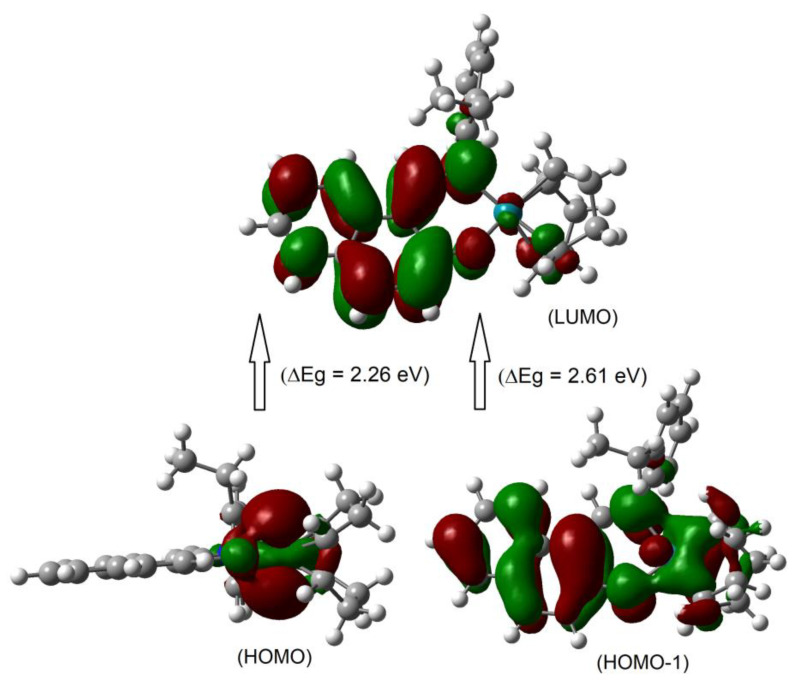
The frontier HOMO-1, HOMO and LUMO orbitals for compound **2** calculated at B3LYP/SDD with PCM in chloroform.

Although the metal centred d–d (MM) electron transitions for the diamagnetic closed shell transition metals like Rh(I) and Zn(II) are not allowed, there were some d-electron clouds in the HOMO/HOMO–1. This is due to identical symmetry of the metal-d MO and ligand MOs, which provides a small amount of the electron clouds to the metal-d MO (Figure 4 and Figure S8). In fact, a very small amount of the electron clouds was observed in the LUMO, which reflected minor back donation again due to identical symmetry. As a result, excited state properties possibly deliver a very small d–d (MM) contribution in addition to the metal–ligand and/or ligand–ligand (ML/LL) π-transitions (Figure 4 and Figure S8 and Table 2) [19,23,25,26]. The frontier HOMO-1, HOMO and LUMO are presented in Figure 4 and Figure S8. The HOMO comprised mostly metal-d_z_^2^ electron moieties, while the HOMO-1 comprised metal-d_z_^2^, sal/naphthal-π and (η^4^-cod)-π electron moieties. The LUMO comprised sal/naphthal-σ and -π with a small number of metal-d_xy_ electrons moieties. The energy gap for HOMO to LUMO transitions is considerably low (ΔE = 2.43 (**1**) or 2.26 (**2**) eV) and results in the highest MO contributions (e.g., 98 or 97%) to the excitation protocol.

### 2.2. X-ray Analyses 

**Figure 5:**
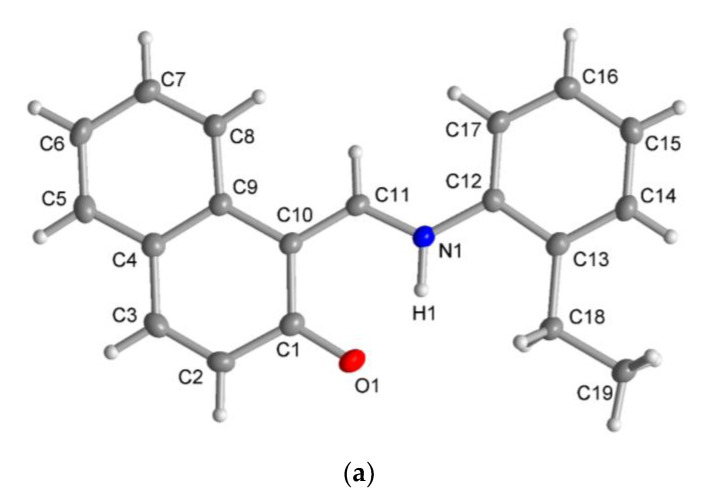

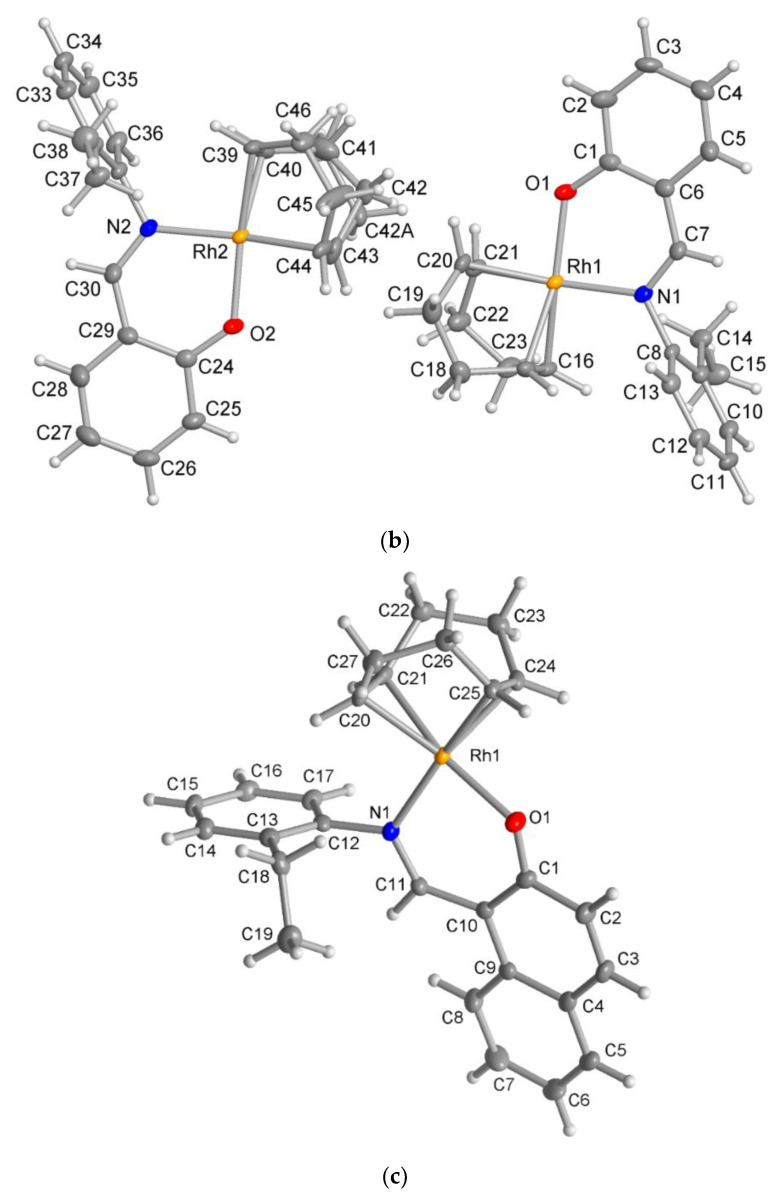
Molecular structures for compounds HL^2^ (**a**), **1** (**b**) and **2** (**c**) (50% thermal ellipsoids). There is a disorder in the cod ring in the Rh2 molecule in **1**.

The X-ray molecular structure revealed that the ligand HL^2^ exists as the zwitterionic (imine)N-H^+^···^–^O(phenol) (ketoamine form) in the solid-state (Figure 5a), which is occasionally seen in Schiff base compounds [27]. The N-H proton could be found and refined. The molecular packing in HL^2^ is organized by a π-π and a C-H···π contact (Figure S4). The molecular structures for the rhodium complex **1** or **2** demonstrated that the deprotonated Schiff base ligand (L^1^ or L^2^) coordinated to the Rh(η^4^-cod)-fragment as a six-membered N,O-chelate ligand to the rhodium atom with a close-to-square-planar geometry if one considers the midpoints of the cod double bonds and the N,O donor atoms (Figure 5b,c). 

There are two symmetry-independent molecules in the asymmetric unit in compound **1**. This included a molecule with Rh1 and a molecule with Rh2 (Figure 5b), as reported in the analogous Rh(η^4^-cod)-Schiff base complexes [17,19]. Two symmetry-independent molecules or two identical chemical formula units in the asymmetric unit [28] define a structure with Z′ = 2, where Z′ is the number of formula units in the unit cell (which is eight in **1**) divided by the number of independent general positions (four in **1**) [29]. Structures with Z′ > 1 [30] can signal a metastable structure [28,29,30,31,32,33], two co-existing conformations of very similar energy in a molecule [34,35,36] or originate from special supramolecular interactions among or between the symmetry-independent units [37,38,39,40,41,42].

**Figure 6:**
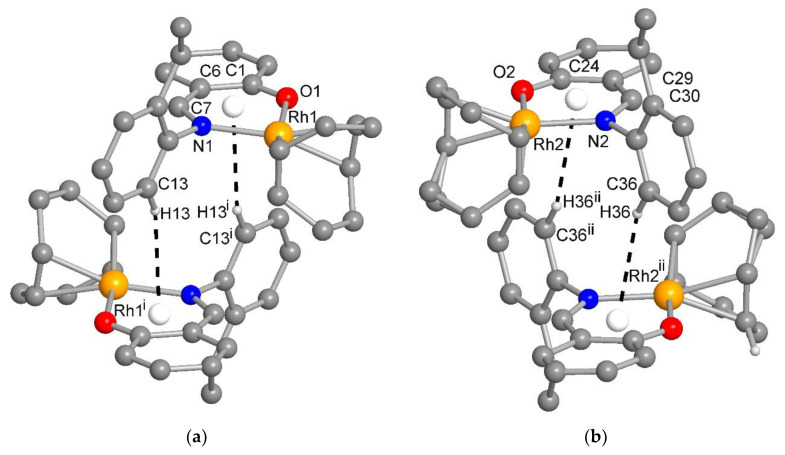
The reciprocal or pairwise C-H···metallochelate-π contacts between a pair of each of the symmetry-independent molecules in **1** with Rh1 (**a**) and Rh2 (**b**) (distance and angle details are given in Table S1). For clarity, the H atoms were omitted except for those of the C-H···π contact. Symmetry transformation i = –x, 1–y, 1–z; ii = 2–x, 1–y, 2–z.

The molecular packing in **1** and **2** was largely controlled by van der Waals interactions between the C-H groups. There were no π-π interactions and only two recognized C-H···π contacts each between the molecules in **1** and **2** (Figures S5 and S6). Remarkably, in **1,** there were reciprocal or pairwise C-H···π contacts between a pair of each of the symmetry-independent molecules (Figure 6). Both C-H···π contacts originated from the ortho-C-H atom of the ethylphenyl ring and pointed onto the six-membered Rh-N^O chelate ring. This metallacycle can be regarded as metalloaromatic according to Masui, who had proposed an electron delocalization within a metal–heterocyclic chelate ring so that it exhibited metalloaromaticity [43,44,45,46,47,48]. This pairwise C-H contact to the Rh-N^O chelate ring could be a reason for the two symmetry-independent molecules in **1**. In **2,** the C-H···π contacts were a normal interaction between two naphthyl C-H atoms onto either the naphthyl or the ethylphenyl ring (Figure S6).

The coordination around Rh in the molecular structure of **1** or **2** was also seen in the closely related structures of Rh(η^4^-cod)-complexes with the chiral/achiral-Schiff base ligands [5,6,7,8,9,10,17,18,19,20]. Selected bond lengths and angles for HL^2^, **1** and **2** are listed in Table 3. They were comparable to the analogous Rh(η^4^-cod)-Schiff bases complexes [5,6,7,8,9,10,17,18,19,20]. The Rh−C (cod-ligand) bond lengths in **1** or **2** were slightly different, which reflected the fact that the Rh atom was bound asymmetrically to the C=C olefinic carbon atoms *trans* to the nitrogen or oxygen donor atoms. The structures for **1** and **2** were optimized at B3LYP/SDD (Figure S7) and provided similar results as the X-ray molecular structures (Table 3).

### 2.3. Keto-Enol Tautomerism 

**Scheme 3:**
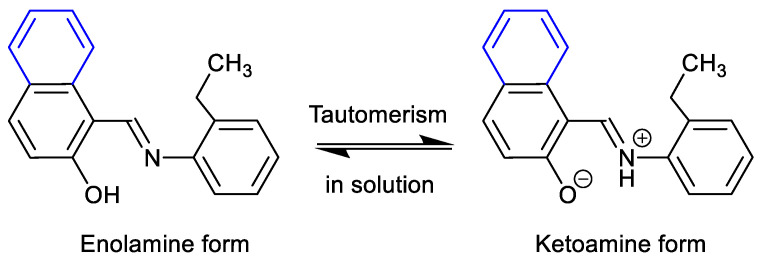
Keto-enol tautomerism of HL^2^ in solution.

To check the existence of keto-enol tautomerism (i.e., keto ⇆ enol equilibrium) in the solution (Scheme 3) [24,48], we ran ^1^H NMR spectra for HL^1^ and HL^2^ in CD_3_OD and DMSO-d_6_ in addition to CDCl_3_ (Figure S3 and Table 1). A significant chemical shift downfield by ca. 0.12 ppm (CDCl_3_) and 0.30 ppm (DMSO-d_6_) for C*H*=N and ca. 0.44 ppm (DMSO-d_6_) for OH was observed with increasing solvent polarity from CDCl_3_ to CD_3_OD to DMSO-d_6_ for HL^2^. Similarly, the C*H*=N peak shifted downfield by 0.28 ppm (DMSO-d_6_) for HL^1^. The C*H*=N peak appeared in duplicate with a separation of 18.0 Hz in CD_3_OD, which corresponded to the presence of both the keto- and enol forms with an equilibrium of almost equimolar amounts in solution. However, the peak for OH (enol form) and/or NH (keto form) was not seen in CD_3_OD due to rapid proton-exchange with the alcoholic group (OD). In DMSO-d_6_, the spectrum also showed two peaks (separated by ca. 5.0 Hz) for C*H*=N or OH/NH. The exchange of the phenolic proton (OH) between the oxygen and nitrogen atoms is considerably slow on the ^1^H NMR time scale, which results in the detection of both signals for keto- and enol-forms in CD_3_OD and DMSO-d_6_, which stabilize the ionic ketoamine form. 

### 2.4. Phase Transformation and Thermal Stability

**Figure 7:**
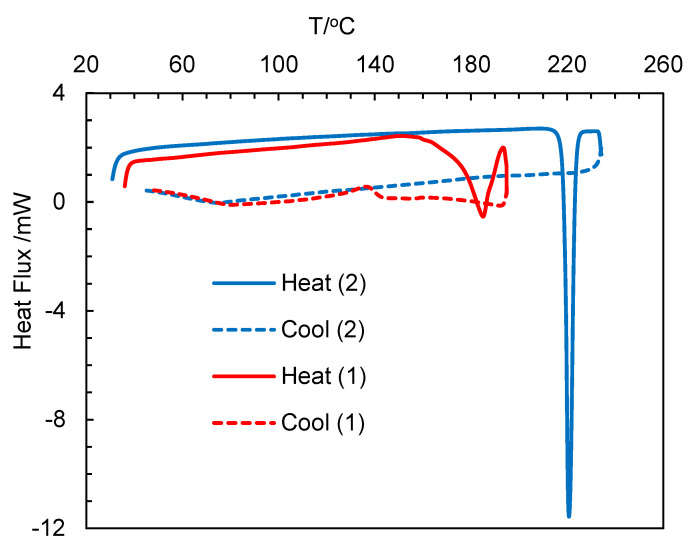
Differential scanning calorimetry (DSC) curves for compounds **1** and **2**.

The differential scanning calorimetry (DSC) curves for HL^2^, **1** and **2** are show in Figure 7 (Figure S10) and data are listed in Table 4. The heating curves showed an endothermic peak with a considerable amount of heat of transformation (ΔH/kJ mol^−1^), which corresponded to a phase transformation from the crystalline-solid to isotropic-liquid phase and subsequently confirmed the thermal stability of the compounds, as reported for the analogous Rh(η^4^-cod)-Schiff base complexes [19]. The cooling curves showed no peak on the reverse direction, suggesting an irreversible phase transformation. The repeated heating curves in the second cycle for the same probe reproduced similar peaks for HL^2^, while no peak was observed for **1** or **2**. The phase transformation temperature for **2** (ca. 221 °C) was considerably higher than **1** (ca. 185 °C), which corresponds to higher thermal stability in accordance with the high molecular weight. Similarly, the free ligand (HL^2^) showed a low phase transformation temperature (ca. 92 °C) due to low molecular weight.

## 3. Materials and Methods

### 3.1. Materials and Characterization

All reactions were carried out under dry nitrogen gas using collecting flux. Solvents were dried and redistilled under nitrogen prior to use: benzene over Na metal and methanol over CaO. IR spectra were recorded on a Nicolet iS10 spectrometer as KBr discs at ambient temperature. UV-Vis spectra were measured with a Shimadzu UV 1800 spectrophotometer in chloroform at 25 °C. Differential scanning calorimeter (DSC) analyses were performed on a Shimadzu DSC-60 at the range of 30–240 °C (ca. 5 °C above the corresponding melting point) with a rate of 10 K min^–1^. ^1^H NMR spectra were recorded on a Bruker Avance DPX 400 spectrometer at 20 °C in CDCl_3_, DMSO-d_6_ or CD_3_OD. Electron impact (EI) mass spectra were recorded with a Thermo-Finnigan TSQ 700 mass spectrometer.

### 3.2. Synthesis of the Ligands

2-Hydroxybenzaldehyde (salicylaldehyde: 1.22 g, 10.0 mmol) or 2-hydroxy-1-naphthaldehyde (1.7218 g, 10.0 mmol) was dissolved into 20 mL of ethanol, and 3–4 drops of concentrated H_2_SO_4_ were added into the solution, which was then stirred for ca. 10 min at room temperature. An equimolar amount of 2-ethyl aniline (1.2118 g, 10.0 mmol, dissolved in 5 mL of ethanol) was slowly added to this solution. The reaction mixture was then refluxed for ca. 23 h for 2-hydroxybenzaldehyde and ca. 6 h for 2-hydroxy-1-naphthaldehyde. Thin-layer chromatography (TLC) was run to monitor the progress of the reaction. After completion of the reaction, the volume of the solution was reduced to about 70% by a rotary evaporator. This concentrated solution was left standing in open air for crystallization via slow evaporation of the solvent. A semi-liquid product was obtained after 3–4 days, which was then dried in open air to get brown-yellow (*E*)-2-(((2-ethylphenyl)imino)methyl)phenol (HL^1^). After one day, a precipitate was formed, filtered off and washed three times with ethanol followed by n-hexane (2 mL each). The products were dried in open air to obtain orange-yellow microcrystals of (*E*)-1-(((2-ethylphenyl)imino)methyl)naphthalen-2-ol (HL^2^). X-ray quality single crystals were grown by slow evaporation of a concentrated methanol solution of HL^2^ at room temperature. 

(*E*)-2-(((2-ethylphenyl)imino)methyl)phenol (HL^1^) or (*E*)-1-(((2-ethylphenyl)imino)-methyl)naphthalen-2-ol (HL^2^): Yield: 1.75 g (78%, based on 2-hydroxybenzaldehyde). IR (KBr): ν = 3059, 3017, 2967, 2932 w (H-Ar), 1616, 1595 vs. (C=N) and 1570 vs. (C=C) cm^–1^. UV-Vis (0.10 mM, MeOH): λ_max_/nm (ε_max_/L mol^−1^ cm^−1^) = 341 (7030), 267 (8000) and 227 (12,440). ^1^H NMR (400 MHz, CDCl_3_): δ/ppm **=** 1.28 (t, *J*_HH_ = 7.6 Hz, 3H, CH_3_), 2.84 (q, *J*_HH_ = 7.6 Hz, 2H, CH_2_), 6.99 (t, *J*_HH_ = 7.6 Hz, 1H, H_4_), 7.08 (d, *J*_HH_ = 8.0 Hz, 1H, H_2_), 7.13 (dd, *J*_HH_ = 6.4, 1.6 Hz, 1H, H_12_), 7.27–7.33 (m, 3H, H_10_,_11_,_13_), 7.40–7.46 (m, 2H, H_3,5_), 8.62 (s, 1H, CHN) and 13.47 (br, H, OH/NH) (for hydrogen atom numbering, see Scheme 1).

^1^H NMR (400 MHz, DMSO-d_6_): δ/ppm **=** 1.17 (t, *J*_HH_ = 7.6 Hz, 3H, CH_3_), 2.73 (q, *J*_HH_ = 7.6 Hz, 2H, CH_2_), 6.97–7.02 (m, 2H, H_2,4_), 7.25–7.28 (m, 1H, H_12_), 7.29–7.33 (m, 3H, H_10,11,13_), 7.44 (t, *J*_HH_ = 7.00 Hz, 1H, H_3_), 7.68 (d, *J*_HH_ = 7.6 Hz, 1H, H_5_), 8.90 (s, 1H, CHN) and 13.31 (br, 1H, OH/NH).

(*E*)-1-(((2-ethylphenyl)imino)methyl)naphthalen-2-ol (HL^2^): Yield: 2.20 g (80%, based on 2-hydroxy-1-naphthaldehyde). IR (KBr): ν = 3097, 3034, 2966, 2933 w (H-Ar), 1620, 1597 vs. (C=N) and 1543 vs. (C=C) cm^–1^. UV-Vis (0.10 mM, CHCl_3_): λ_max_/nm (ε_max_/L mol^−1^ cm^−1^) = 442 (6257), 376 (7343) and 318 (8771). ^1^H NMR (400 MHz, CDCl_3_): δ/ppm = 1.34 (t, *J*_HH_ = 7.6 Hz, 3H, CH_3_), 2.90 (q, *J*_HH_ = 7.6 Hz, 2H, CH_2_), 7.17 (d, *J*_HH_ = 9.2 Hz, 1H, H_14_), 7.30 (d, *J*_HH_ = 9.6 Hz, 1H, H_17_), 7.35–7.39 (m, 4H, H_2,6,15,16_), 7.55 (t, *J*_HH_ = 8.0 Hz, 1H, H_7_), 7.74 (d, *J*_HH_ = 8.0 Hz, 1H, H_8_), 7.84 (d, *J*_HH_ = 9.2 Hz, 1H, H_5_), 8.13 (d, *J*_HH_ = 8.4 Hz, 1H, H_3_), 9.34 (s, 1H, CHN) and 15.63 (br, 1H, OH/NH) (for hydrogen atom numbering, see Scheme 1).

^1^H NMR (400 MHz, CD_3_OD): δ/ppm **=** 1.33 (dt, *J*_HH_ = 7.6, 4.0 Hz, 3H, CH_3_), 2.88 (dq, *J*_HH_ = 7.6, 7.2 Hz, 2H, CH_2_), 6.94 (t, *J*_HH_ = 8.8 Hz, 1H, H_16_), 7.24–7.41 (m, 4H, H_2,14,15,17_), 7.51–7.56 (m, 1H, H_6_), 7.64–7.72 (m, 2H, H_7,8_), 7.85 (t, *J*_HH_ = 10 Hz, 1H, H_5_), 8.24–8.29 (m, 1H, H_3_) and 9.46, 9.51 (s, 1H, CHN) (for hydrogen atom numbering, see Scheme 1). 

^1^H NMR (400 MHz, DMSO-d_6_) δ/ppm = 1.24 (t, *J*_HH_ = 7.6 Hz, 3H, CH_3_), 2.81 (q, *J*_HH_ = 7.6 Hz, 2H, CH_2_), 7.04 (d, *J*_HH_ = 9.2 Hz, 1H, H_14_), 7.27 (t, *J*_HH_ = 7.2 Hz, 1H, H_16_), 7.35–7.40 (m, 3H, H_2,15,17_), 7.55 (t, *J*_HH_ = 7.6 Hz, 1H, H_6_), 7.80 (t, *J*_HH_ = 7.6 Hz, 2H, H_7,8_), 7.95 (d, *J*_HH_ = 9.2 Hz, 1H, H_5_), 8.51 (d, *J*_HH_ = 8.4 Hz, 1H, H_3_), 9.65, 9.66 (s, 1H, CHN) and 16.06, 16.07 (s, 1H, OH/NH) (for hydrogen atom numbering, see Scheme 1).

### 3.3. Synthesis of the Complexes

Two equivalents of HL^1^ or HL^2^ (112.6 mg or 137.5 mg, 0.5 mmol) and one equivalent of [Rh(η^4^-cod)(O_2_CMe)]_2_ (134.2 mg, 0.25 mmol) were dissolved in 10 mL of a mixture of C_6_H_6_:MeOH (2:1, *v/v*) under N_2_ gas and were kept stirring at room temperature. The color changed immediately from orange-red to bright yellow, and a precipitate formed within 30 min in the reaction with HL^2^. The solution was stirred for another ca. 6 h at room temperature. The precipitate was collected through filtration, washed three times with methanol (1 mL each) and dried in vacuo at 40 °C to obtain bright yellow microcrystals of [Rh(η^4^-cod)(L^2^)] (**2**). In the reaction with HL^1^, no precipitate was formed after 6 h of stirring, and the solvent was then evaporated to dryness in a rotary evaporator in vacuo at 40 °C. The residue was dissolved in 2 mL of a mixture of C_6_H_6_:MeOH (2:1, *v/v*), stirred for ca. 30 min and again dried in a rotary evaporator in vacuo at 40 °C. This process was repeated three times, and finally, bright yellow microcrystals of [Rh(η^4^-cod)(L^1^)] (**1**) were obtained. Single crystals suitable for X-ray diffraction measurements were grown by slow diffusion of n-hexane into a concentrated dichloromethane solution of **1** or **2** after 3–4 days at room temperature.

[Rh(η^4^-cod)(L^1^)] (**1**): Yield: 0.150 g (70%), based on [Rh(η^4^-cod)(O_2_CMe)]_2_. IR (KBr): ν = 3057, 3017, 2951, 2934 w (H-Ar), 1609, 1586 vs. (C=N) and 1574, 1528 s (C=C) cm^–1^. UV-Vis (0.20 mM, CHCl_3_): λ_max_/nm (ε_max_/L mol^−1^ cm^−1^) = 399 (2099) and 285 sh. ^1^H NMR (400 MHz, CDCl_3_): δ/ppm = 1.33 (t, *J*_HH_ = 7.6 Hz, 3H, CH_3_), 1.77–1.79 (m, 4H, CH_2_cod_exo_), 2.52–2.54 (m, 4H, CH_2_cod_endo_), 2.93 (q, *J*_HH_ = 6.8 Hz, 2H, CH_2_), 4.27 (s, 4H, =CHcod), 7.05–7.07 (m, 1H, H-Ar), 7.21–7.27 (m, 1H, H-Ar), 7.38–7.49 (m, 4H, H-Ar), 7.61–7.65 (m, 2H, H-Ar) and 8.77 (s, 1H, CHN). MS (EI, 70 eV): *m*/*z* (%) = 435 (80) [M]^+^, 325 (100) [M–cod–H_2_]^+^, 295 (20) [M–cod–H_2_–(CH_3_CH_3_)]^+^, 208 (10) [C_6_H_5_CHNHRh]^+^, 192 (7) [HL^1^–NH_2_OH]^+^, 182 (10) [HL^1^–CH_3_CHO+H]^+^, 168 (15) [HL^1^–CH_3_CH_2_CHO+H]^+^, 103 (10) [Rh]^+^ and 77 (5) [C_6_H_5_]^+^. 

[Rh(η^4^-cod)(L^2^)] (**2**): Yield: 0.180 g (74 %), based on [Rh(η^4^-cod)(O_2_CMe)]_2_. IR (KBr): ν = 3050, 3011, 2965, 2926 w (H-Ar), 1616, 1603 vs. (C=N) and 1572, 1533 s (C=C) cm^–1^. UV-Vis (0.08 mM, CHCl_3_): λ_max_/nm (ε_max_/L mol^−1^ cm^−1^) = 417 (2638), 325 (8300) and 275 (9413). ^1^H NMR (400 MHz, CDCl_3_): δ/ppm = 1.35 (t, *J*_HH_ = 7.6 Hz, 3H, CH_3_), 1.77–1.79 (m, 4H, CH_2_cod_exo_), 2.52–2.54 (m, 4H, CH_2_cod_endo_), 2.92 (q, *J*_HH_ = 6.8 Hz, 2H, CH_2_), 4.26 (s, 4H, =CHcod), 7.37–7.43 (m, 4H, H-Ar), 7.55–7.68 (m, 2H, H-Ar), 7.77 (d, *J*_HH_ = 7.6 Hz, 1H, H-Ar), 7.93–7.99 (m, 2H, H-Ar), 8.14 (d, *J*_HH_ = 7.2, Hz, 1H, H-Ar) and 9.36 (s, 1H, CHN). MS (EI, 70 eV): *m*/*z* (%) = 485 (80) [M]^+^, 375 (100) [M–cod–H_2_]^+^, 343 (15) [M–cod–H_2_–(CH_3_CH_3_)]^+^, 275 (15) [HL^2^]^+^, 242 (10) [HL^2^–NH_2_OH]^+^, 218 (40) [L^2^–CH_3_CH_2_CHO]^+^, 105 (10) [C_6_H_5_CH_2_CH_3_–H]^+^, 103 (7) [Rh]^+^ and 77 (10) [C_6_H_5_]^+^. 

### 3.4. Computational Method

Computations were performed with the Gaussian 09 program package [49]. The initial geometries for optimization were generated from the X-ray structures of compounds **1** and **2**, respectively. The initial geometry was optimized with B3LYP/3-21G, which was then reoptimized with B3LYP/SDD [19,23,50,51,52]. For calculations of excited state properties, time-dependent density functional theory (TD-DFT) was employed with different combinations of the functionals B3LYP and M06 and the basis sets SDD and DEF2SVP. The simulated spectra thus obtained were very similar with little shifting of band maxima and were also comparable with the experimental spectra (Figure S9). These results further suggest the validity and reliability of the methods employed. The PCM (Polarization Continuum Model) in chloroform and 72 excited states (roots) were considered for the calculations (Tables S2 and S3) [19,23]. Assessments of excited state properties and molecular orbitals (MOs) calculations were carried out at the same level of theory. The simulated spectra were generated with the software SpecDis (version 1.71) [53,54] applying the Gaussian band shape with an exponential half-width σ = 0.16 eV.

### 3.5. X-ray Structure Determination

Suitable crystals were carefully selected under a polarized light microscope covered in protective oil and mounted on a cryo-loop. The single crystal diffraction data was collected using a Rigaku XtaLAB Synergy S four circle diffractometer with a Hybrid Pixel Array Detector and a PhotonJet X-ray source for Cu-Kα radiation (λ = 1.54184 Å) with a multilayer mirror monochromator. Data collection at 100.0 ± 0.1 K using ω-scans. Data reduction and absorption correction were performed with CrysAlisPro 1.171.41.105a [55]. The structures were solved using direct methods (SHELXT-2015), and full-matrix least-squares refinements on F^2^ were carried out using the SHELXL-2017/1 program package in OLEX 2.1.3 [56,57,58]. All hydrogen atoms on C were positioned geometrically (with C–H = 0.95 Å for aromatic and aliphatic CH, C–H = 1.00 Å for ternary CH_,_ C–H = 0.99 Å for CH_2_ and C–H = 0.98 Å for CH_3_) and were refined using riding models (AFIX 43, 13, 23 and 137 with U_iso(H)_ = 1.2 U_eq_(CH, CH_2_) and 1.5 U_eq_(CH_3_). The protic hydrogen atom for NH in HL^2^ was found and refined freely. Crystal data and details on the structure refinement are given in Table 5. Graphics were drawn with the program DIAMOND [59]. Computations on the supramolecular interactions were carried out with PLATON for Windows [60,61,62]. The CCDC numbers 2201154-2201156 for HL^2^, **1** and **2**, respectively, contain the supplementary crystallographic data reported in this paper. These data can be obtained free of charge from the Cambridge Crystallographic Data Centre via www.ccdc.cam.ac.uk/data_request/cif.

## 4. Conclusions

The mononuclear complexes of [Rh(η^4^-cod)(L^1^)] (**1**) and [Rh(η^4^-cod)(L^2^)] (**2**) were synthesized from the Schiff bases of (*E*)-2-(((2-ethylphenyl)imino)methyl)phenol (HL^1^) and (*E*)-1-(((2-ethylphenyl)imino)methyl)naphthalen-2-ol (HL^2^), respectively. The X-ray structure showed the ligand HL^2^ to exist as the zwitterionic (imine)N-H^+^···^–^O(phenol) (ketoamine form) instead of usual (imine)N···H-O(phenol) (enolamine form) in the solid-state, and it displayed keto-enol tautomerism in solution as evidenced by ^1^H NMR studies. The structure for **1** or **2** confirmed coordination of the deprotonated Schiff base to the Rh(η^4^-cod)-fragment as a six-membered N^O-chelate around the Rh(I) with a close-to-square-planar geometry. There are two symmetry-independent molecules (with Rh1 and Rh2) in the asymmetric unit in **1**, which gives a structure with Z′ = 2. The crystal packing analysis revealed both π-π and C-H···π contacts in HL^2^, while there were only two C-H···π contacts in **1** or **2**. The reciprocal or pairwise C-H···π contacts (i.e., C-H to Rh-N^O metalloaromatic-ring) between a pair of each of the symmetry-independent molecules could be the reason for the two symmetry-independent molecules in **1**.

## Figures and Tables

**Table 1:** ^1^H NMR (400 MHz) data for the Schiff bases and complexes at 20 °C.

Entity	Solvents	Assignments						
		CH_3_	CH_2_	CH_2_cod_exo_/ CH_2_cod_endo_	CHcod	CHN	OH/NH	H-Ar
HL^1^	CDCl_3_	1.28 (t, *J*_HH_ = 7.6 Hz)	2.84 (q, *J*_HH_ = 7.6 Hz)			8.62 (s)	13.47 (br)	6.99 (t, *J*_HH_ = 7.6 Hz, 1H, H_4_), 7.08 (d, *J*_HH_ = 8.0 Hz, 1H, H_2_), 7.13 (dd, *J*_HH_ = 6.4, 1.6 Hz, 1H, H_12_), 7.27–7.33 (m, 3H, H_10_,_11_,_13_), 7.40–7.46 (m, 2H, H_3,5_)
	DMSO-d_6_	1.17 (t, *J*_HH_ = 7.6 Hz)	2.73 (q, *J*_HH_ = 7.6 Hz)			8.90 (s)	13.31 (br)	6.97–7.02 (m, 2H, H_2,4_), 7.25–7.28 (m, 1H, H_12_), 7.29–7.33 (m, 3H, H_10,11,13_), 7.44 (t, *J*_HH_ = 7.0 Hz, 1H, H_3_), 7.68 (d, *J*_HH_ = 7.6 Hz, 1H, H_5_)
HL^2^	CDCl_3_	1.34 (t, *J*_HH_ = 7.6 Hz)	2.90 (q, *J*_HH_ = 7.6 Hz)			9.34 (s)	15.63 (br)	7.17 (d, *J*_HH_ = 9.2 Hz, 1H, H_14_), 7.30 (d, *J*_HH_ = 9.6 Hz, 1H, H_17_), 7.35–7.39 (m, 4H, H_2_,_6,15,16_), 7.55 (t, *J*_HH_ = 8.0 Hz, 1H, H_7_), 7.74 (d, *J*_HH_ = 8.0 Hz, 1H, H_8_), 7.84 (d, *J*_HH_ = 9.2 Hz, 1H, H_5_), 8.13 (d, *J*_HH_ = 8.4 Hz, 1H, H_3_)
	CD_3_OD	1.33 (m)	2.88 (m)			9.46, 9.51 (18.0 Hz)		6.94 (t, *J*_HH_ = 8.8 Hz, 1H, H_16_), 7.24–7.41 (m, 4H, H_2,14,15,17_), 7.51–7.56 (m, 1H, H_6_), 7.64–7.72 (m, 2H, H_7,8_), 7.85 (t, *J*_HH_ = 10.0 Hz, 1H, H_5_), 8.24–8.29 (m, 1H, H_3_)
	DMSO-d_6_	1.24 (t, *J*_HH_ = 7.6 Hz)	2.81 (q, *J*_HH_ = 7.6 Hz)			9.65, 9.66 (5.0 Hz)	16.06, 16.07 (5.0 Hz)	7.04 (d, *J*_HH_ = 9.2 Hz, 1H, H_14_), 7.27 (t, *J*_HH_ = 7.2 Hz, 1H, H_16_), 7.35–7.40 (m, 3H, H_2,15,17_), 7.55 (t, *J*_HH_ = 7.6 Hz, 1H, H_6_), 7.80 (t, *J*_HH_ = 7.6 Hz, 2H, H_7,8_), 7.95 (d, *J*_HH_ = 9.2 Hz, 1H, H_5_), 8.51 (d, *J*_HH_ = 8.4 Hz, 1H, H_3_)
1	CDCl_3_	1.33 (t, *J*_HH_ = 7.6 Hz)	2.93 (q, J_HH_ = 6.8 Hz)	1.77–1.79/2.52–2.54 (m)	4.27 (s)	8.77 (s)		7.05–7.07 (m, 1H, H-Ar), 7.21–7.27 (m, 1H, H-Ar), 7.38–7.49 (m, 4H, H-Ar), 7.61–7.65 (m, 2H, H-Ar)
2	CDCl_3_	1.35 (t, *J*_HH_ = 7.6 Hz)	2.92 (q, *J*_HH_ = 6.8 Hz)	1.77–1.79/2.52–2.54 (m)	4.26 (s)	9.36 (s)		7.37–7.43 (m, 4H, H-Ar), 7.55–7.68 (m, 2H, H-Ar), 7.77 (d, *J*_HH_ = 7.6 Hz, 1H, H-Ar), 7.93–7.99 (m, 2H, H-Ar), 8.14 (d, *J*_HH_ = 7.2, Hz, 1H, H-Ar)

s = singlet, d = doublet, t = triplet, dd = doublet of doublets, dt = doublet of triplets, m = multiplet.

**Table 2:** Selected excited state properties and assignments for **1** and **2**, calculated at B3LYP/SDD with PCM in chloroform.

Wavelength (nm) ^a^	Excited States	Oscillator Strength	Electronic Transition (MOs Contribution, %) ^b^	Assignments ^c^
	Compound **1**			
475	1	0.0022	H→L (98)	MM, ML
402 (399)	2	0.0426	H-1→L (71)	MM, ML
361	4	0.0217	H-3→L (42), H-2→L (54)	MM, ML, LL
335 (ca. 310 sh)	7	0.1432	H-3→L (54), H-2→L (38)	MM, ML, LL
291	12	0.1853	H-1→L + 1 (18), H-1→L + 2 (42)	MM, ML, LL
246	29	0.2134	H-2→L + 1 (12), H-1→L + 6 (32)	MM, ML, LL
228	41	0.1113	H-4→L + 1 (12), H→L + 9 (11)	ML, LL
212	55	0.2316	H-7→L + 1 (17), H-1→L + 11 (12)	MM, LL
	Compound **2**			
487	1	0.0030	H→L (97)	MM, ML
410 (417)	2	0.0662	H-1→L (76)	MM, ML, LL
348	6	0.2166	H-3→L (40), H-2→L (52)	MM, ML, LL
311 (323)	10	0.2079	H-1→L+1 (53)	MM, ML, LL
285 (275)	17	0.0700	H-1→L+3 (26), H→L+6 (22)	MM, ML, LL
249	33	0.1012	H-3→L+1 (19), H-3→L + 3 (28)	MM, ML, LL
233	44	0.1513	H-4→L+1 (9), H-4→L + 3 (36)	ML, LL
215	65	0.2176	H-4→L+5 (11), H-3→L + 11 (11)	ML, LL

^a^ Experimental values are in parentheses; ^b^ H/L = HOMO/LUMO and MOs = molecular orbitals; ^c^ MM = metal centered d–d transitions; ML = metal–ligand/ligand–metal charge transfer transitions, LL = intra-ligand charge transfer transitions.

**Table 3:** Selected bond lengths [Å] and angles [°] in the X-ray and optimized structures for HL^2^, **1** and **2**.

Compound HL^2^		Compound 1					Compound 2		
	X-ray	X-ray (with Rh1)		Opt. Str.	X-ray (with Rh2)			X-ray	Opt. Str.
C1-O1 C11-N1 C12-N1 N1-H1 C11-H11 C1-C10 C10-C11 O1-C1-C10 C11-N1-C12 C10-C11-N1 C11-N1-H1 C12-N1-H1	1.270 (2) 1.324 (2) 1.413 (1) 0.95 (2) 0.950 (0) 1.450 (2) 1.387 (2) 122.3 (1) 126.3 (1) 124.0 (1) 113.0 (1) 121.0 (1)	Rh1–O1 Rh1–N1 Rh1–C16 Rh1–C17 Rh1–C20 Rh1–C21 C1-O1 C7-N1 C8-N1 C16-C17 C20-C21 O1-Rh-N1 O1-Rh-C16 O1-Rh-C17 O1-Rh-C20 O1-Rh-C21 N1-Rh-C16 N1-Rh-C17 N1-Rh-C20 N1-Rh-C21	2.037 (2) 2.080 (2) 2.113 (2) 2.118 (2) 2.130 (2) 2.139 (2) 1.300 (3) 1.298 (3) 1.450 (3) 1.404 (3) 1.391 (3) 90.35 (7) 159.16 (8) 160.29 (8) 84.98 (8) 87.92 (8) 94.21 (8) 96.48 (8) 157.90 (8) 163.56 (8)	2.044 2.099 2.155 2.175 2.174 2.195 1.328 1.327 1.456 1.425 1.416 89.75 157.71 161.19 84.71 87.37 95.98 98.23 157.07 164.21	Rh2–O2 Rh2–N2 Rh2–C39 Rh2–C40 Rh2–C43 Rh2–C44 C24-O2 C30-N2 C31-N2 C39-C40 C43-C44 O2-Rh-N2 O2-Rh-C39 O2-Rh-C40 O2-Rh-C43 O2-Rh-C44 N2-Rh-C39 N2-Rh-C40 N2-Rh-C43 N2-Rh-C44	2.026 (2) 2.084 (2) 2.124 (2) 2.119 (2) 2.137 (2) 2.129 (2) 1.301 (3) 1.295 (3) 1.450 (3) 1.402 (3) 1.386 (4) 90.44 (7) 162.34 (8) 156.99 (8) 85.59 (8) 86.34 (8) 95.04 (8) 96.61 (8) 163.79 (8) 157.51 (8)	Rh1–O1 R1h–N1 Rh1–C20 Rh1–C21 Rh1–C24 Rh1–C25 C1-O1 C11-N1 C12-N1 C20-C21 C24-C25 O1-Rh-N1 O1-Rh-C20 O1-Rh-C21 O1-Rh-C24 O1-Rh-C25 N1-Rh-C20 N1-Rh-C21 N1-Rh-C24 N1-Rh-C25	2.025 (2) 2.076 (3) 2.112 (3) 2.133 (4) 2.126 (3) 2.148 (4) 1.294 (4) 1.307 (5) 1.456 (5) 1.401 (5) 1.388 (5) 88.70 (1) 153.10 (1) 166.40 (1) 87.40 (1) 86.50 (1) 97.60 (1) 96.70 (1) 152.00 (1) 168.70 (1)	2.048 2.087 2.160 2.181 2.181 2.205 1.329 1.332 1.461 1.423 1.415 88.35 157.72 162.05 86.19 88.65 96.48 98.25 156.56 164.91

Opt. Str. = Optimized structure at B3LYP/SDD.

**Table 4:** Phase transformation and thermal stability for the compounds.

Compounds	Peaks Temperature/°C (Heating Curve)	ΔH (kJ mol^–1^)
HL^2^	92 (1st cycle)	−19.93
	89 (2nd cycle)	−15.60
**1**	185 (1st cycle)	−19.77
**2**	221 (1st cycle)	−37.57

**Table 5:** Crystal data and structure refinement for HL^2^, **1** and **2**.

	HL^2^	1	2
Formula	C_19_H_17_NO	C_23_H_26_NORh	C_27_H_28_NORh
*M*_r_	275.33	435.36	485.41
Cryst. size, mm^3^	0.17 × 0.20 × 0.81	0.06 × 0.07 × 0.14	0.04 × 0.03 × 0.12
Crystal system	monoclinic	monoclinic	monoclinic
Temp. (K)	100	100	100
Space group	*I*2/a	*P*2_1_/n	*P*2_1_/c
*a*, Å	14.4394 (2)	10.6023 (1)	10.5105 (2)
*b*, Å	5.92690 (10)	18.6289 (1)	7.44870 (10)
*c*, Å	33.3937 (5)	19.2399 (1)	27.3231 (5)
*β*, deg	102.4790 (10)	98.696 (1)	94.400 (2)
*V*, Å^3^	2790.35 (7)	3756.37 (5)	2132.81 (6)
Z	8	8	4
*D*_calcd_, g cm^−3^	1.311	1.540	1.512
*μ*, mm^−1^ *θ* range (°)	0.630 2.7–67.1	7.425 3.2–67.1	6.606 3.2–78.4
*F*(000)	1168	1792	1000
Trans. (max/min)	0.979/0.979	0.662/0.642	0.930/0.930
*hkl* range	±17; ±7; ±39	12; 22; 22	±12; ±8; ±32
Refl. measured	23976	51759	24582
Refl. unique	2492	6705	3812
*R*_int_	0.0436	0.0322	0.0382
Param. refined/restraints	195/0	481/0	272/0
GoF (*F*^2^) ^a^	1.048	1.048	1.249
*R*_1_/*wR*_2_ [I>2σ (I)] ^b^	0.0377/0.1003	0.0226/0.0570	0.0358/0.0764
*R*_1_/*wR*_2_ (all data) ^b^	0.0400/0.1022	0.0246/0.0579	0.0374/0.0770
Max./min. Δ*ρ* (e. Å^−3^) ^c^	0.192/–0.222	0.576/–0.635	0.818/–0.875
CCDC number	2201154	2201155	2201156

^a^ Goodness-of-fit = [∑[w(Fo2 − Fc2)2]/(n − p)]1/2; ^b^ R1 = [∑(||Fo| − |Fc||)/∑|Fo|]; wR2 = [∑[w(Fo2 − Fc2)2]/∑[w(Fo2)2]]1/2; ^c^ Largest difference peak and hole.

## Data Availability

The data presented in this study are available on request from the corresponding authors.

## Supplementary Materials

The following supporting information can be downloaded at: https://www.mdpi.com/article/10.3390/molecules28010172/s1, Figure S1. EI-mass spectra for complexes [Rh(η^4^-cod)(L^1^)] (**1**) (**a**) and [Rh(η^4^-cod)(L^2^)] (**2**) (**b**). Figure S2. ^1^H NMR spectrum for compound **1** in CDCl_3_ at 20 °C. EI-MS for **1** and **2.** Figure S3. ^1^H NMR spectra for HL^1^ in DMSO-d_6_ (**a**) and for HL^2^ in CD_3_OD (**b**) and DMSO-d_6_ (**c**) at 20 °C. Figure S4. Sections of the packing diagram in HL^2^ along (**a**) a, (**b**) b and (**c**) presentation of the π-π and a C-H···π contact. Figure S5. Section of the packing diagram in **1** along *a*. Figure S6. Sections of the packing diagram in **2** along *a* (**a**) and *b* (**b**). Figure S7. Optimized structures for compounds **1** (**a**) and **2** (**b**) at B3LYO/SDD. Figure S8. The frontier HOMO-1, HOMO and LUMO orbitals for compound **1** calculated at B3LYP/SDD with PCM in chloroform. Figure S9. Simulated spectra for **2** with different combinations of the functionals and the basis sets (with PCM in chloroform). Gaussian band shape with exponential half-width s = 0.16 eV. Experimental spectrum for **2** (0.08 mM) in chloroform. Figure S10. Differential scanning calorimetry (DSC) curves for HL2. Table S1. Distance and angle details for the pairwise C-H···metallochelate-π contacts in **1**. Table S2. Distance and angle details for C-H···π contacts in **2**. Table S3. List of excited states, excitation energy (eV), wavelength (nm), oscillator strength (f) and MO contributions for compound **1** at B3LYP/SDD with PCM in chloroform. Table S4. List of excited states, excitation energy (eV), wavelength (nm), oscillator strength (f) and MO contributions for compound **2** at B3LYP/SDD with PCM in chloroform.

## Sample Availability

Not available.
