# Supplementary material for: Schiff Base in Ketoamine Form and Rh(η4-cod)-Schiff Base Complex with Z′ = 2 Structure from Pairwise C-H···Metallochelate-π Contacts
Source: Molecules. 2022 Dec 25;28(1):172. doi: 10.3390/molecules28010172 (PMC9821875; doi:10.3390/molecules28010172)
Supplement: Supplementary file 1 [file molecules-28-00172-s001.zip › molecules-2082335-supplementary.pdf]

Schiff-base in ketoamine form and Rh(η^4-cod)-Schiff base complex with *Z*' = 2 structure from pairwise C-H···metallochelate-π contacts

Mohammed Enamullah [1,*], Imdadul Haque [1], Amina Khan Resma [1], Dennis Woschko [2] and Christoph Janiak [2,*]

[1] Department of Chemistry, Jahangirnagar University, Dhaka-1342, Bangladesh
[2] Institut für Anorganische Chemie und Strukturchemie, Universität Düsseldorf, Universitätsstr. 1, D-40225 Düsseldorf, Germany
* Correspondence: enamullah@juniv.edu (M.E); janiak@uni-duesseldorf.de (C.J.).

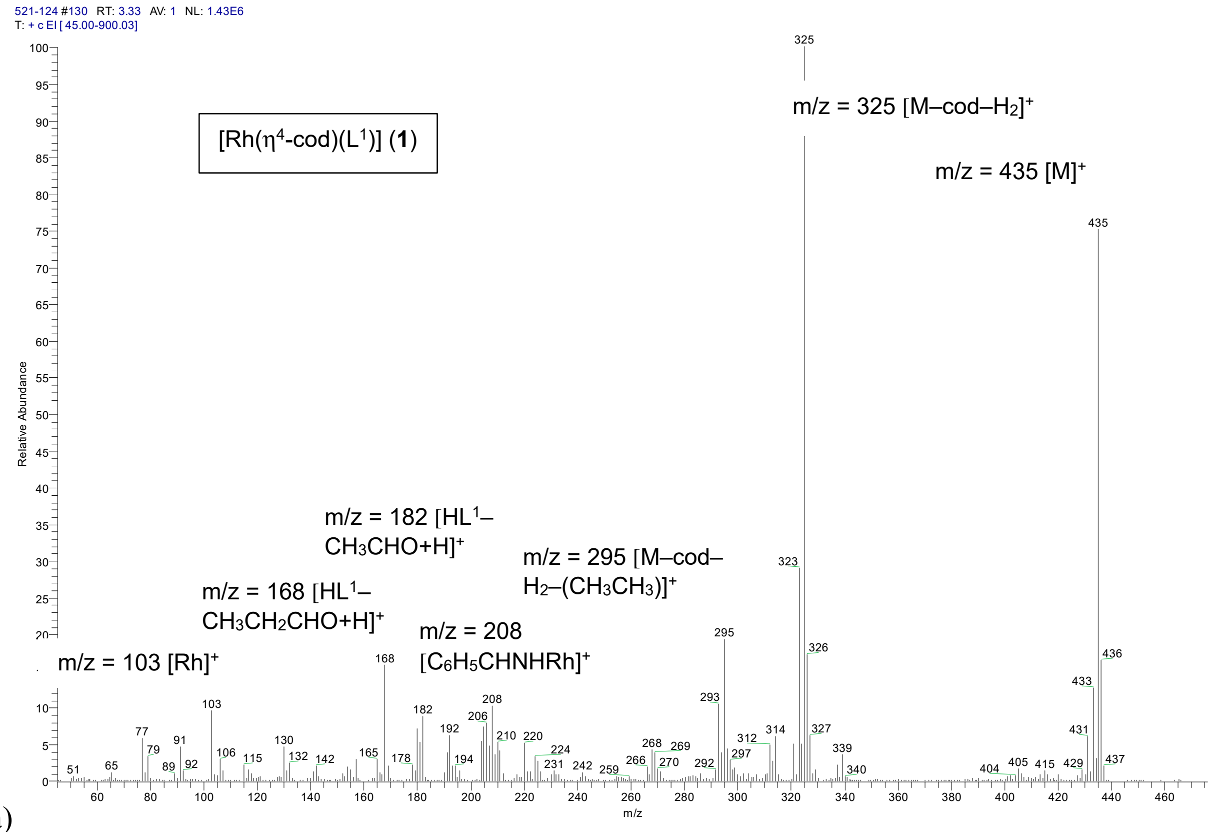

(a)

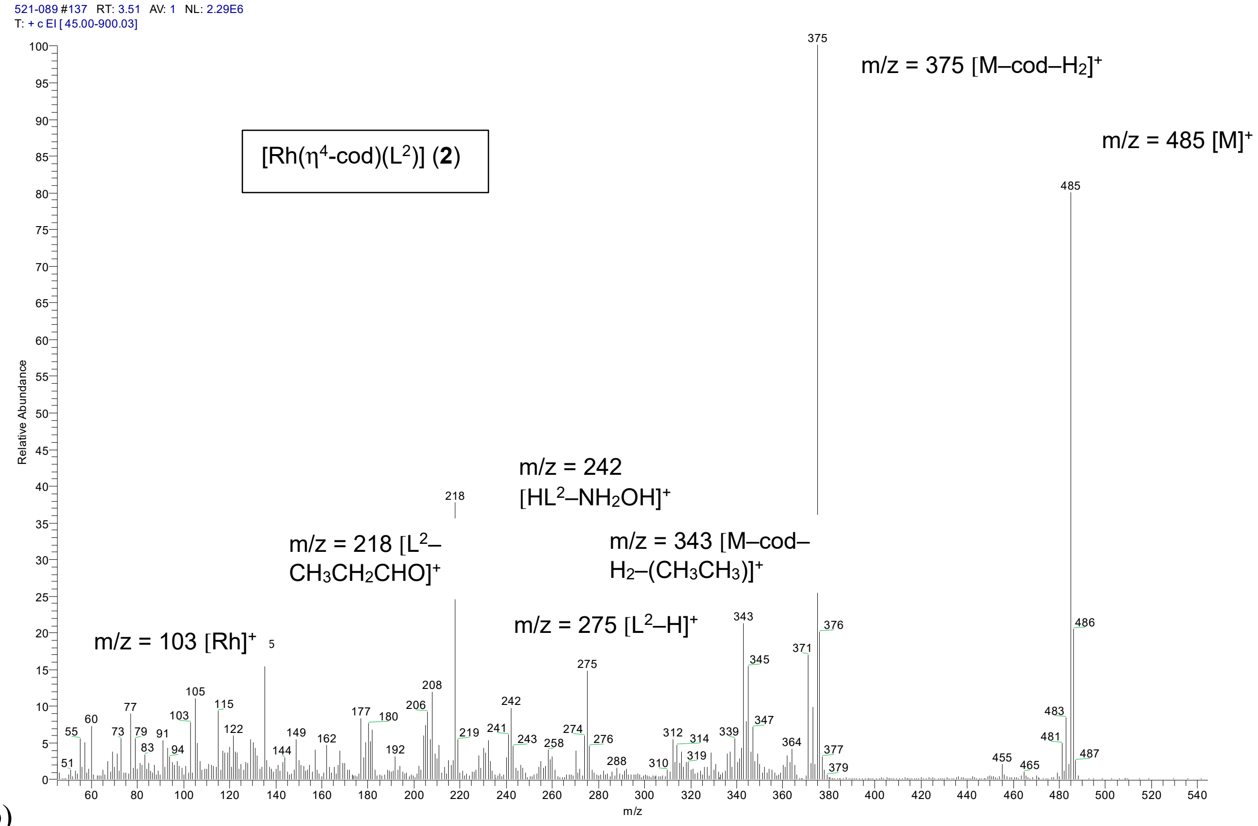

(**b**)

Figure S1. EI-mass spectra for complexes [Rh(η^4-cod)(L^1)] (**1**) (**a**) and [Rh(η^4-cod)(L^2)] (**2**) (**b**).

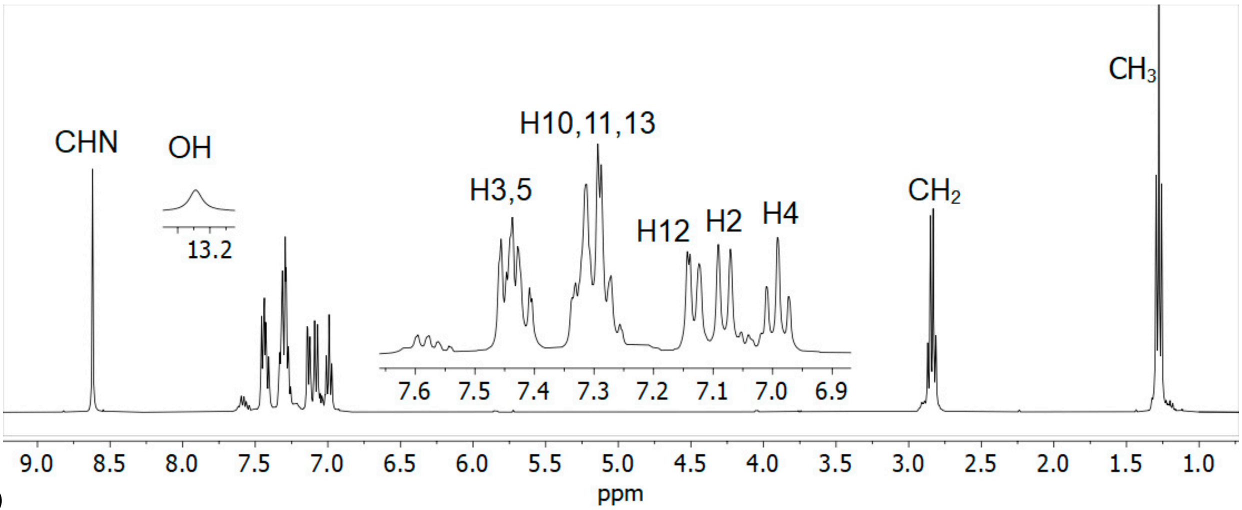

(**a**)

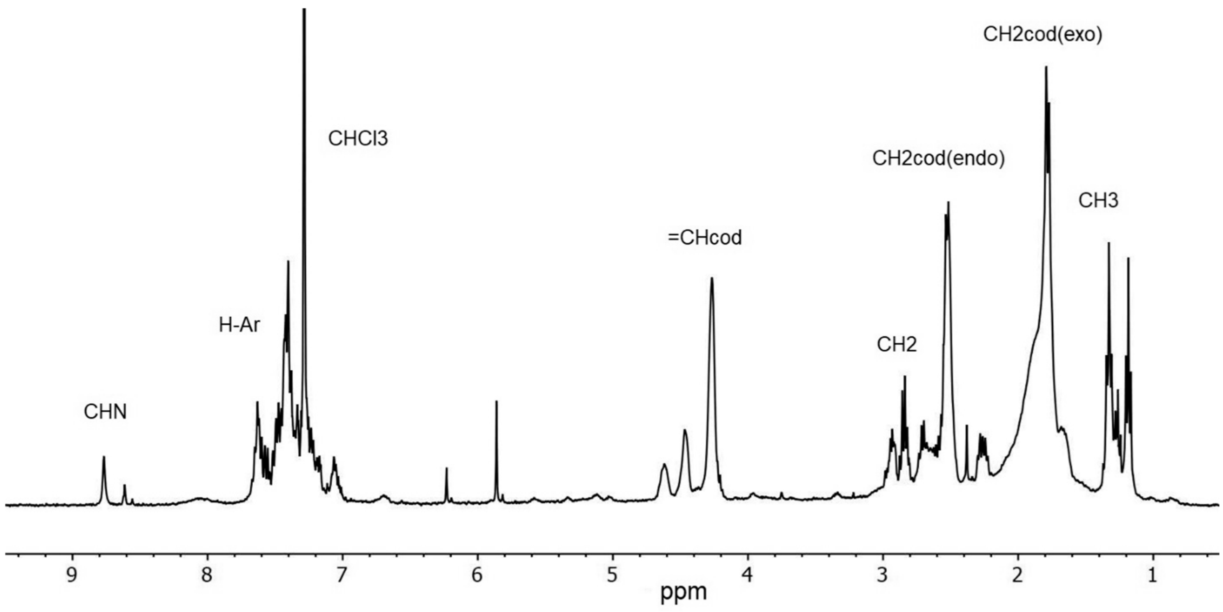

(**b**)

Figure S2. ^{1}H NMR spectra of the Schiff base ligand HL^1 (**a**) and compound **1** (**b**) in $CDCl_3$ at 20 °C.

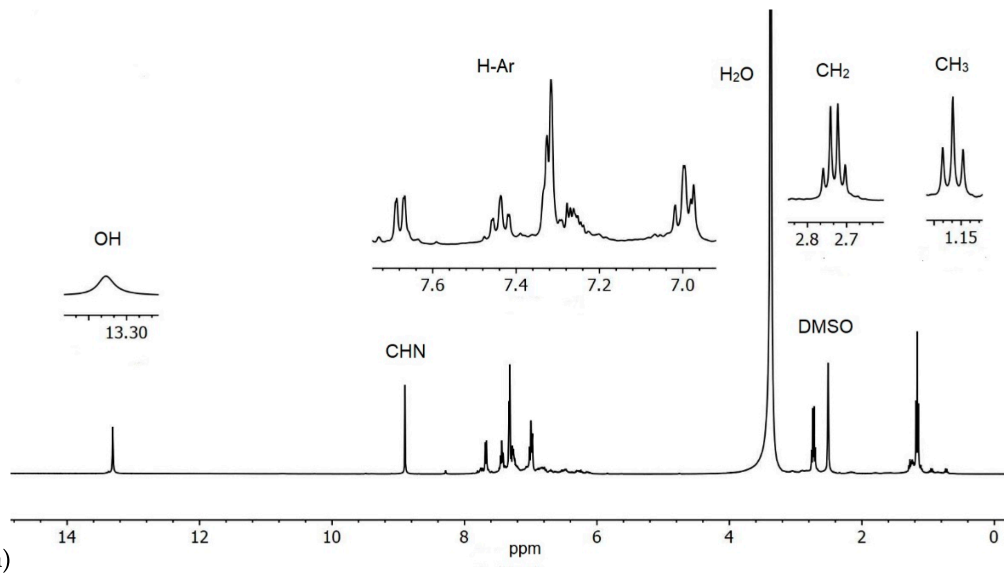

(**a**)

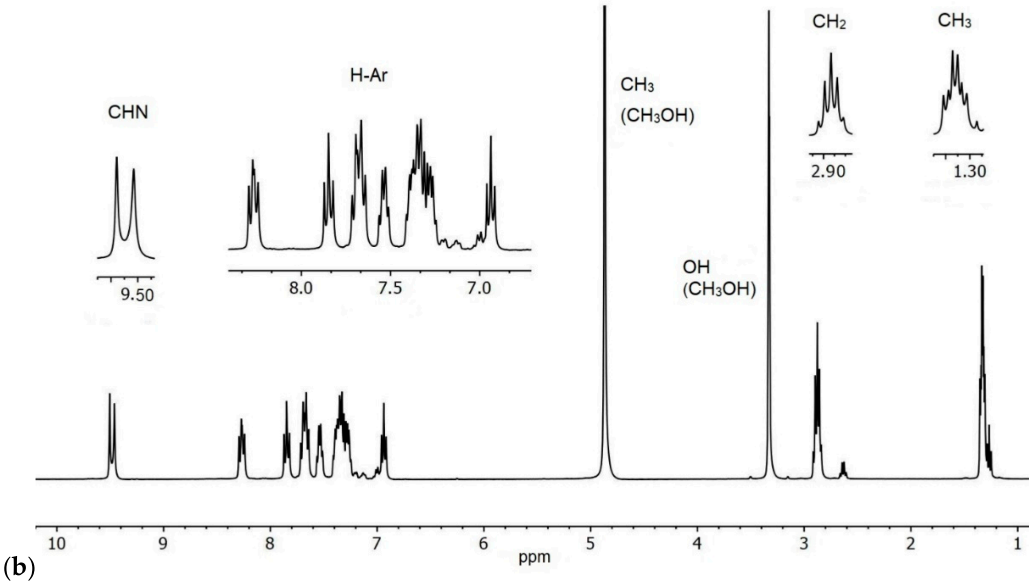

(**b**)

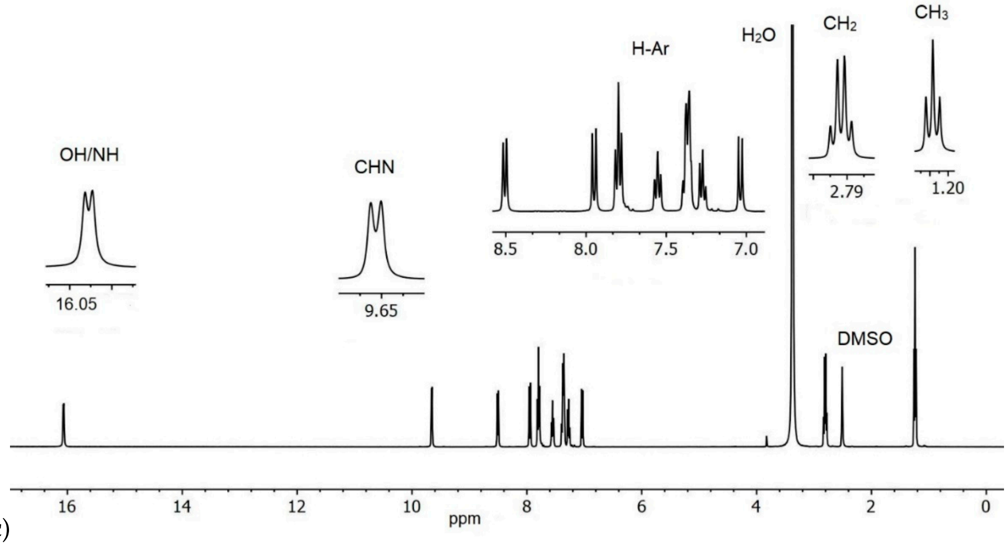

(**c**)

Figure S3. ^{1}H NMR spectra for HL[1] in DMSO-d$_6$ (**a**), and for HL[2] in CD_3OD (**b**) and DMSO-d$_6$ (**c**) at 20 °C.

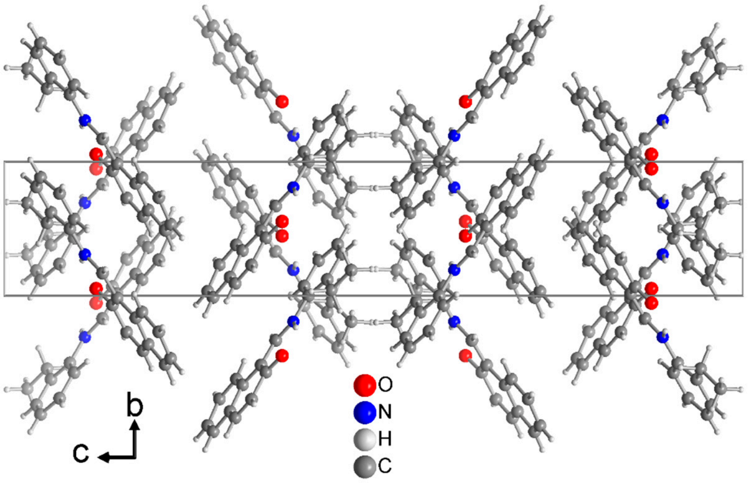

(**a**)

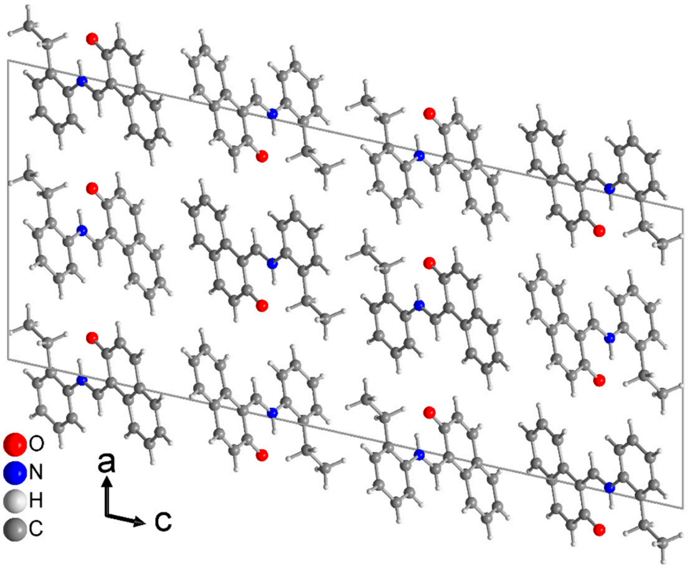

(**b**)

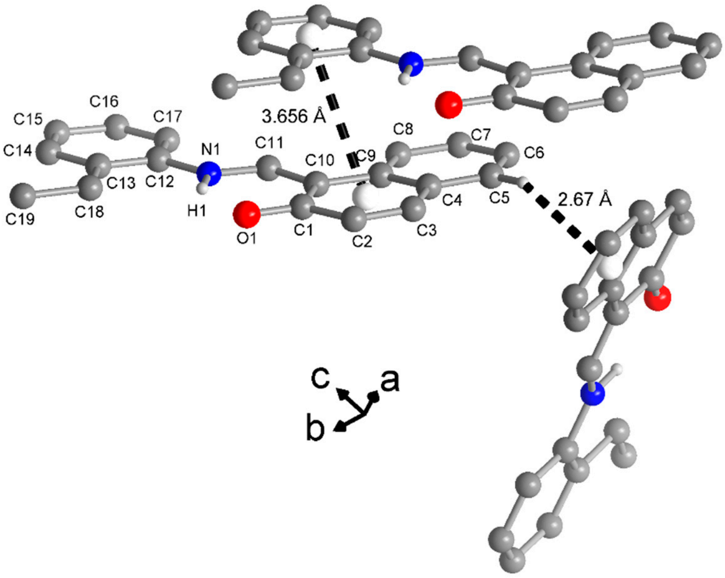

(**c**)

Figure S4. Sections of the packing diagram in HL^2 along (**a**) a, (**b**) b and (**c**) presentation of the π-π and a C-H···π contact.

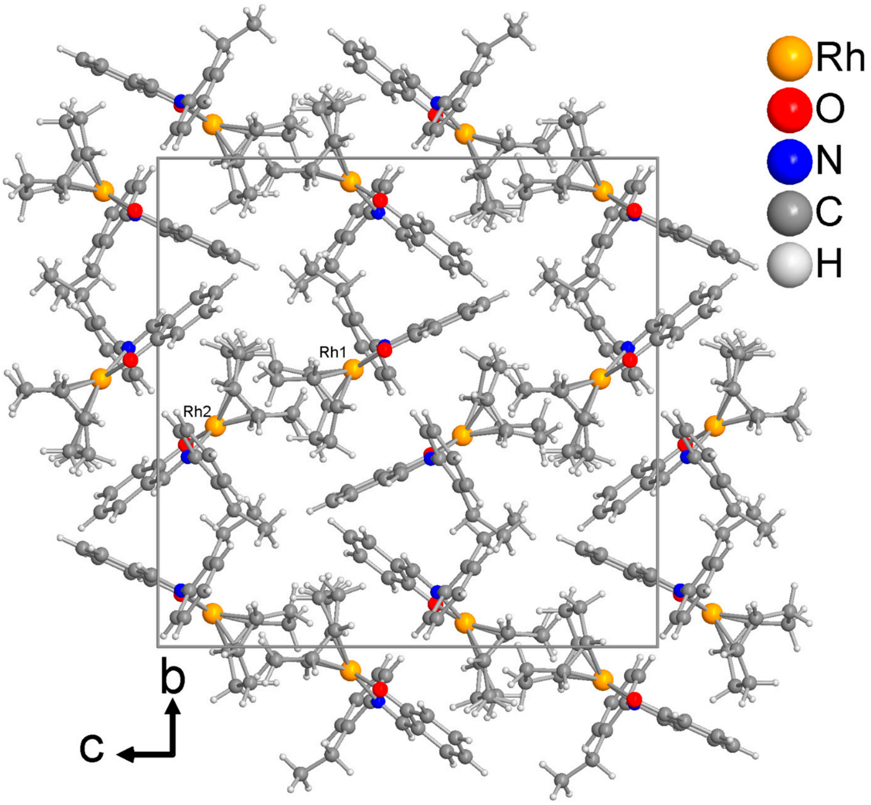

Figure S5. Section of the packing diagram in **1** along *a*. The packing is largely controlled by van der Waals interactions between the C-H groups. There are no π–π interactions. The reciprocal or pairwise C-H···metallochelate-π contacts between a pair of each of the symmetry-independent molecules is shown in Figure 6 in the main text, distance and angle details are given in Table S1.

Table S1. Distance and angle details for the pairwise C-H···metallochelate-π contacts in **1**.

==

Analysis of X-H...Cg(Pi-Ring) Interactions (H..Cg < 3.0 Ang. - Gamma < 30.0 Deg)

==

- Cg(J) = Center of gravity of ring J (Plane number above)
- H-Perp = Perpendicular distance of H to ring plane J
- Gamma = Angle between Cg-H vector and ring J normal
- X-H..Cg = X-H-Cg angle (degrees)
- X..Cg = Distance of X to Cg (Angstrom)
- X-H, Pi = Angle of the X-H bond with the Pi-plane (i.e.' Perpendicular = 90 degrees, Parallel = 0 degrees)

X--H(I)	Res(I)	Cg(J)	[ARU(J)]	H..Cg	H-Perp	Gamma	X-H..Cg	X..Cg	X-H,Pi
C(13) -H(13)	[2]	-> Cg(17)	[3566.02]	2.80	-2.67	17.64	139	3.574(2)	62
C(36) -H(36)	[1]	-> Cg(6)	[3767.01]	2.85	2.67	20.61	139	3.622(3)	57

[3566] = -X,1-Y,1-Z
[3767] = 2-X,1-Y,2-Z

Cg(I) refer to
Cg(17) = Rh1-O1-N1-C1-C6-C7
Cg(6) = Rh2-O2-N2-C24-C29-C30

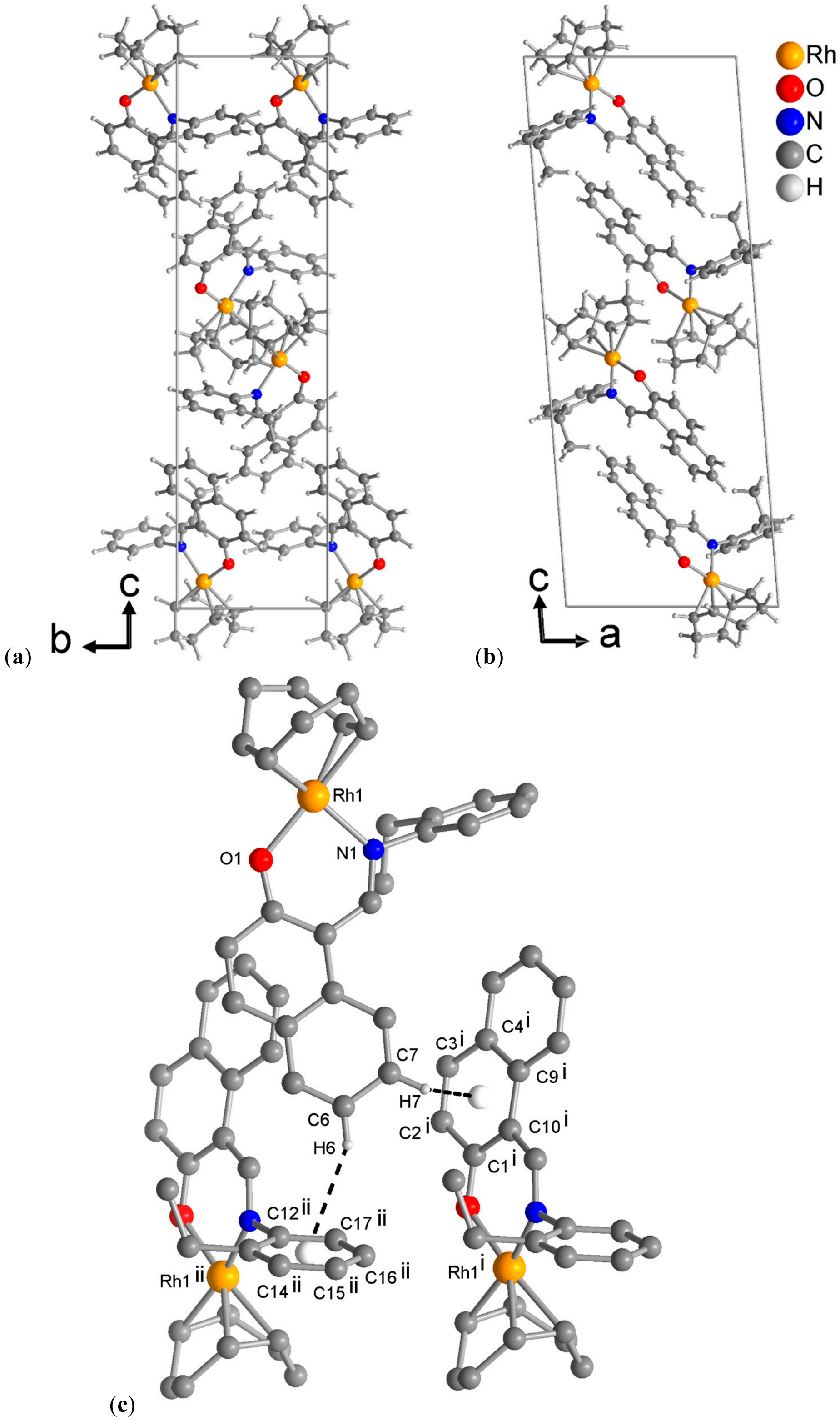

Figure S6. Sections of the packing diagram in **2** along *a* (**a**) and *b* (**b**). The packing is largely controlled by van der Waals interactions between the C-H groups. There are no π–π interactions and only two weak C-H···π contacts (**c**) (distance and angle details are given in Table S2). For clarity the H atoms have been omitted except for those of the C-H···π contact. Symmetry transformation i = 1–x, 1/2+y, 1/2–z; ii = 1–x, –1/2+y, 1/2–z.

Table S2. Distance and angle details for C-H···π contacts in **2**.

==

Analysis of X-H...Cg(Pi-Ring) Interactions (H..Cg < 3.0 Ang. - Gamma < 30.0 Deg)

==

- Cg(J) = Center of gravity of ring J (Plane number above)
- H-Perp = Perpendicular distance of H to ring plane J
- Gamma = Angle between Cg-H vector and ring J normal
- X-H..Cg = X-H-Cg angle (degrees)
- X..Cg = Distance of X to Cg (Angstrom)
- X-H, Pi = Angle of the X-H bond with the Pi-plane (i.e.' Perpendicular = 90 degrees, Parallel = 0 degrees)

X--H(I)	Res(I)	Cg(J)	[ARU(J)]	H..Cg	H-Perp	Gamma	X-H..Cg	X..Cg	X-H,Pi
C(6) -H(6)	[1]	-> Cg(12)	[2645.01]	2.95	-2.83	16.38	145	3.767(4)	65
C(7) -H(7)	[1]	-> Cg(10)	[2655.01]	2.95	-2.79	18.44	115	3.461(4)	43

[2645] = 1-X,-1/2+Y,1/2-Z
[2655] = 1-X,1/2+Y,1/2-Z

The Cg(I) refer to
Cg(10) = C1-C2-C3-C4-C9-C10
Cg(12) = C12-C13-C14-C15-C16-C17

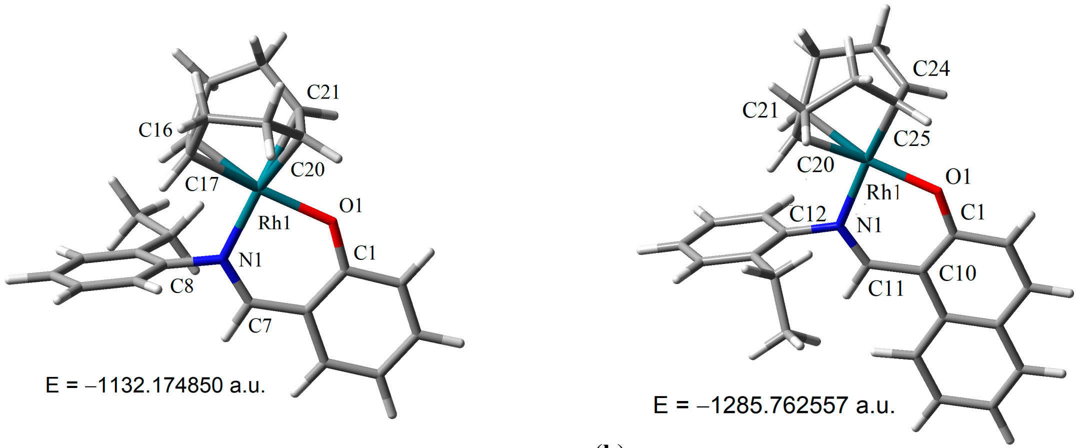

(**a**) (**b**)

Figure S7. Optimized structures for compounds **1** (**a**) and **2** (**b**) at B3LYP/SDD.

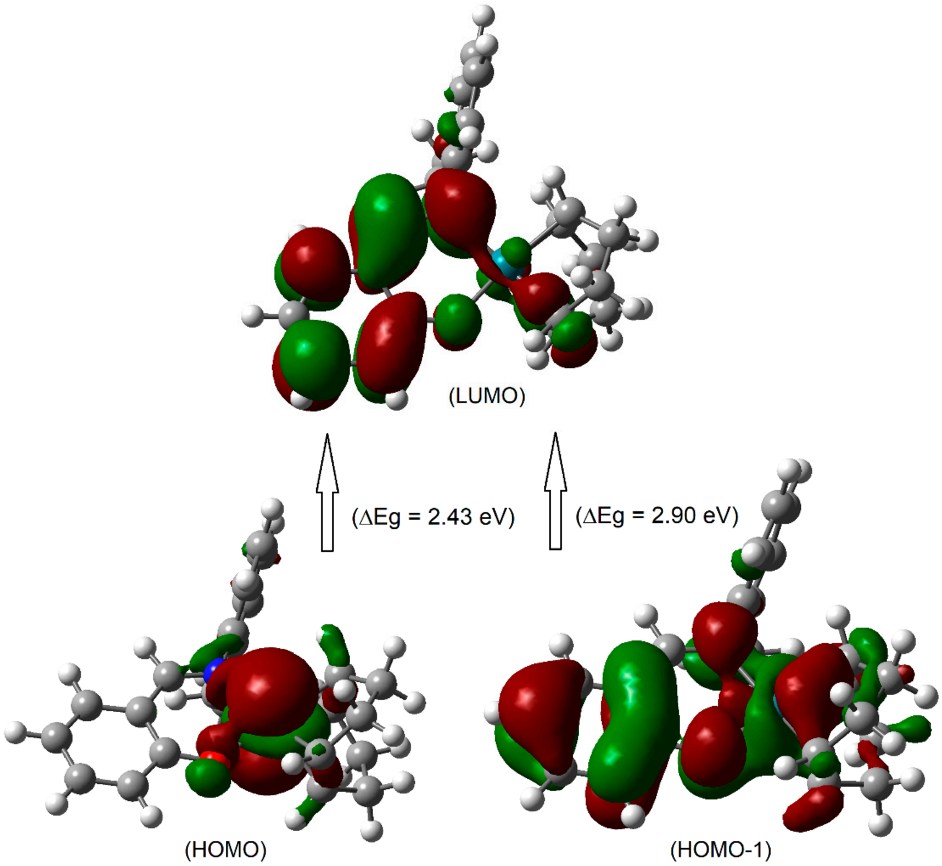

Figure S8. The frontier HOMO-1, HOMO and LUMO orbitals for compound **1** calculated at B3LYP/SDD with PCM in chloroform.

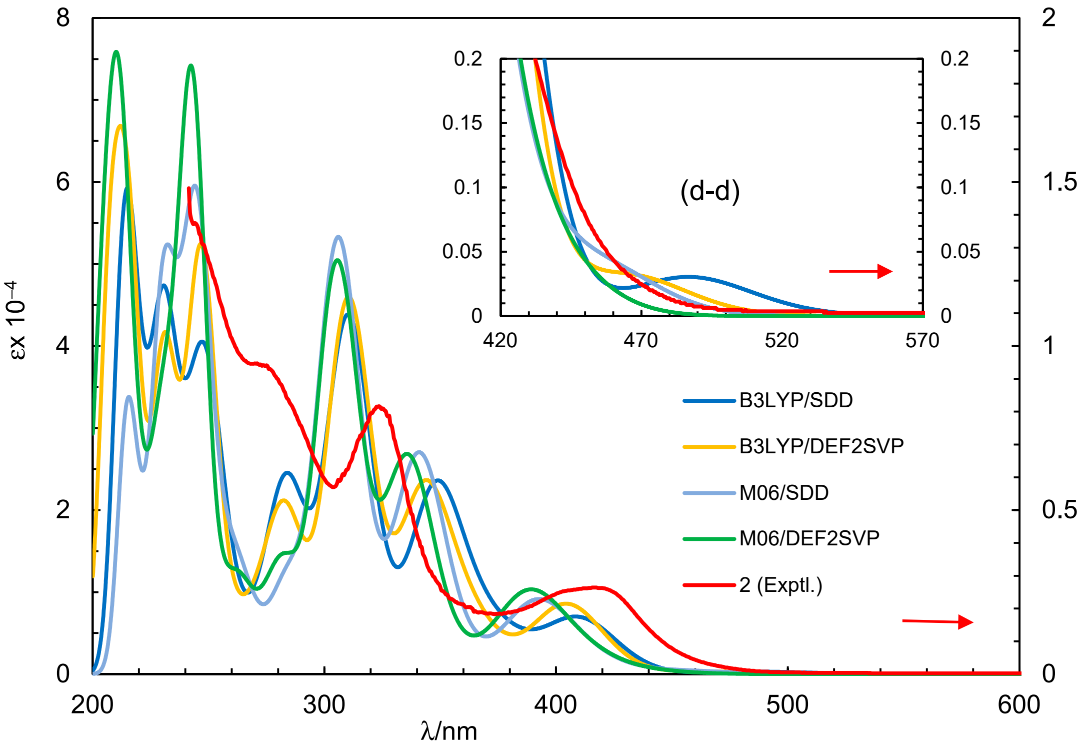

Figure S9. Simulated spectra for **2** with different combinations of the functionals and the basis sets (with PCM in chloroform). Gaussian band shape with exponential half-width s = 0.16 eV. Experimental spectrum for **2** (0.08 mM) in chloroform.

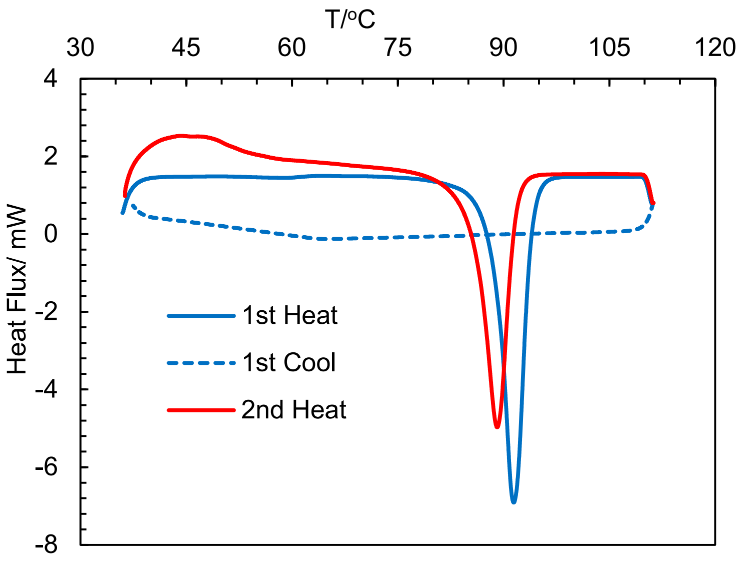

Figure S10. Differential scanning calorimetry (DSC) curves for HL^2.

Table S3. List of excited states, excitation energy (eV), wavelength (nm), oscillator strength (f) and MO contributions for compound **1** at B3LYP/SDD with PCM in chloroform.

```
Excited State   1:      Singlet-A      2.6104 eV  474.95 nm  f=0.0022  <S**2>=0.000
     98 -> 99         0.70043
 This state for optimization and/or second-order correction.
 Total Energy, E(TD-HF/TD-KS) =  -1132.47281265
 Copying the excited state density for this state as the 1-particle RhoCI density.

 Excited State   2:      Singlet-A      3.0848 eV  401.92 nm  f=0.0426  <S**2>=0.000
     97 -> 99         0.59652
     98 ->100         0.20673
     98 ->101         0.17261
     98 ->103         0.17668
     98 ->104        -0.11336

 Excited State   3:      Singlet-A      3.3023 eV  375.44 nm  f=0.0187  <S**2>=0.000
     97 -> 99        -0.34727
     98 ->100         0.37459
     98 ->101         0.27950
     98 ->103         0.27958
     98 ->104        -0.18020
     98 ->105        -0.14663

 Excited State   4:      Singlet-A      3.4360 eV  360.84 nm  f=0.0217  <S**2>=0.000
     95 -> 99        -0.45893
     96 -> 99         0.51955

 Excited State   5:      Singlet-A      3.4716 eV  357.13 nm  f=0.0134  <S**2>=0.000
     98 ->100        -0.33129
     98 ->101         0.57712

 Excited State   6:      Singlet-A      3.6652 eV  338.27 nm  f=0.0055  <S**2>=0.000
     95 ->100         0.32474
     95 ->101         0.18857
     95 ->103         0.28197
     95 ->104        -0.17559
     95 ->105        -0.15418
     96 -> 99        -0.12461
     96 ->100        -0.25078
     96 ->101        -0.17108
     96 ->103        -0.21289
     96 ->104         0.14154
     96 ->105         0.11137

 Excited State   7:      Singlet-A      3.6990 eV  335.18 nm  f=0.1432  <S**2>=0.000
     95 -> 99         0.51865
     96 -> 99         0.43713

 Excited State   8:      Singlet-A      3.8982 eV  318.06 nm  f=0.0028  <S**2>=0.000
```

94 ->100 0.12869
94 ->101 0.11082
94 ->103 0.11257
97 ->100 0.43353
97 ->101 0.30533
97 ->103 0.26656
97 ->104 -0.16031
97 ->105 -0.12649

Excited State 9: Singlet-A 4.0326 eV 307.45 nm f=0.0269 <S**2>=0.000
94 -> 99 0.67100

Excited State 10: Singlet-A 4.0731 eV 304.40 nm f=0.0052 <S**2>=0.000
93 -> 99 0.34296
95 ->101 0.32823
95 ->103 0.13498
95 ->104 -0.10344
96 ->100 0.34022
96 ->103 0.20411
96 ->104 -0.12736
96 ->105 -0.11518

Excited State 11: Singlet-A 4.0792 eV 303.94 nm f=0.0073 <S**2>=0.000
93 -> 99 0.58990
95 ->101 -0.23569
96 ->100 -0.16096
96 ->103 -0.10561

Excited State 12: Singlet-A 4.2596 eV 291.07 nm f=0.1853 <S**2>=0.000
94 -> 99 -0.10245
96 ->101 -0.15153
97 ->100 -0.30351
97 ->101 0.45896
98 ->100 -0.19183
98 ->102 -0.21927
98 ->103 0.15743

Excited State 13: Singlet-A 4.2728 eV 290.17 nm f=0.0643 <S**2>=0.000
97 ->100 -0.18375
97 ->101 0.28818
98 ->100 0.34074
98 ->102 0.35204
98 ->103 -0.30998
98 ->104 0.10829
98 ->105 0.11471

Excited State 14: Singlet-A 4.3256 eV 286.63 nm f=0.0139 <S**2>=0.000
96 ->101 -0.11915
98 ->100 -0.19109
98 ->101 -0.13132

98 ->102 0.55929
98 ->103 0.29453
98 ->104 -0.11650
98 ->105 -0.11031

Excited State 15: Singlet-A 4.3987 eV 281.86 nm f=0.0175 <S**2>=0.000
91 -> 99 -0.10798
92 -> 99 0.23643
95 ->100 0.21579
95 ->101 -0.12310
96 ->101 0.50984
97 ->100 -0.11133
98 ->103 0.14866

Excited State 16: Singlet-A 4.4356 eV 279.52 nm f=0.0025 <S**2>=0.000
91 -> 99 -0.30143
92 -> 99 0.54920
96 ->101 -0.21986

Excited State 17: Singlet-A 4.5581 eV 272.01 nm f=0.0246 <S**2>=0.000
91 -> 99 0.31480
92 -> 99 0.21727
95 ->100 -0.21735
95 ->101 0.30644
96 ->100 -0.16960
96 ->101 0.13490
98 ->103 0.16484
98 ->104 0.26105
98 ->109 -0.12964

Excited State 18: Singlet-A 4.5636 eV 271.68 nm f=0.0023 <S**2>=0.000
91 -> 99 -0.26735
92 -> 99 -0.19133
97 ->100 0.25036
97 ->101 0.11523
97 ->102 0.21189
97 ->103 -0.22000
97 ->104 0.11752
98 ->103 0.16384
98 ->104 0.32747
98 ->109 -0.17650

Excited State 19: Singlet-A 4.5834 eV 270.50 nm f=0.0116 <S**2>=0.000
90 -> 99 -0.18552
91 -> 99 0.30455
92 -> 99 0.17555
95 ->101 -0.13699
97 ->100 0.13803
97 ->102 0.47525
97 ->103 -0.14640

```
     98 ->104       -0.11371

 Excited State  20:      Singlet-A      4.5967 eV  269.72 nm  f=0.0100  <S**2>=0.000
     90 -> 99       -0.14857
     91 -> 99        0.15764
     92 -> 99        0.10324
     95 ->100        0.21746
     95 ->101       -0.27382
     96 ->100        0.17295
     96 ->101       -0.12327
     97 ->102       -0.25156
     98 ->104        0.34672
     98 ->105       -0.11543
     98 ->109       -0.14257

 Excited State  21:      Singlet-A      4.6230 eV  268.19 nm  f=0.0034  <S**2>=0.000
     97 ->100       -0.22550
     97 ->101       -0.18042
     97 ->102        0.39142
     97 ->103        0.36818
     97 ->104       -0.12348
     97 ->105       -0.11389
     98 ->104        0.19848

 Excited State  22:      Singlet-A      4.6572 eV  266.22 nm  f=0.0003  <S**2>=0.000
     98 ->103        0.21411
     98 ->105        0.62717

 Excited State  23:      Singlet-A      4.8014 eV  258.23 nm  f=0.0005  <S**2>=0.000
     97 ->103       -0.16644
     98 ->103        0.16365
     98 ->104        0.23861
     98 ->107       -0.27937
     98 ->108       -0.12496
     98 ->109        0.48621

 Excited State  24:      Singlet-A      4.8432 eV  256.00 nm  f=0.1657  <S**2>=0.000
     89 -> 99        0.11057
     90 -> 99        0.51792
     91 -> 99        0.21153
     97 ->103       -0.14316
     97 ->104       -0.27649
     97 ->105        0.14888

 Excited State  25:      Singlet-A      4.8746 eV  254.35 nm  f=0.0044  <S**2>=0.000
     89 -> 99        0.65941
     90 -> 99       -0.10339
     91 -> 99        0.14486

 Excited State  26:      Singlet-A      4.9135 eV  252.34 nm  f=0.0446  <S**2>=0.000
```

90 -> 99 0.17046
94 ->100 -0.10874
97 ->103 0.36158
97 ->104 0.31925
97 ->105 0.36070
98 ->109 0.12086

Excited State 27: Singlet-A 4.9985 eV 248.04 nm f=0.0238 <S**2>=0.000
93 ->100 0.17642
93 ->101 0.14996
93 ->103 0.11375
94 ->100 0.27691
94 ->101 0.22835
94 ->103 0.15165
96 ->100 -0.11783
96 ->103 0.10462
97 ->104 0.40785

Excited State 28: Singlet-A 5.0342 eV 246.28 nm f=0.0876 <S**2>=0.000
90 -> 99 -0.12899
93 ->100 0.11053
94 ->100 0.16042
94 ->103 0.10358
96 ->100 0.26302
96 ->102 0.36032
96 ->103 -0.23966
97 ->105 0.32613

Excited State 29: Singlet-A 5.0474 eV 245.64 nm f=0.2134 <S**2>=0.000
90 -> 99 -0.21151
94 ->100 0.11029
96 ->100 -0.24688
96 ->102 -0.21359
96 ->103 0.21116
97 ->104 -0.23122
97 ->105 0.39990

Excited State 30: Singlet-A 5.0932 eV 243.43 nm f=0.0085 <S**2>=0.000
96 ->100 -0.19906
96 ->101 -0.14083
96 ->102 0.52666
96 ->103 0.31173
96 ->104 -0.13119
96 ->105 -0.11392

Excited State 31: Singlet-A 5.1799 eV 239.36 nm f=0.0278 <S**2>=0.000
92 ->100 0.24209
92 ->101 0.10907
93 ->100 -0.14086
93 ->102 -0.24172

94 ->100 0.19089
94 ->101 -0.12089
94 ->102 0.10750
95 ->100 0.20696
95 ->102 0.37676
95 ->103 -0.13364
96 ->102 0.15237

Excited State 32: Singlet-A 5.2006 eV 238.40 nm f=0.0163 <S**2>=0.000
93 ->100 0.12853
93 ->101 -0.14108
94 ->100 0.13605
94 ->101 -0.27172
98 ->106 0.54808

Excited State 33: Singlet-A 5.2140 eV 237.79 nm f=0.0481 <S**2>=0.000
93 ->100 -0.17976
93 ->101 0.14580
94 ->100 -0.15603
94 ->101 0.35473
95 ->102 0.15304
98 ->106 0.39347

Excited State 34: Singlet-A 5.2492 eV 236.20 nm f=0.0011 <S**2>=0.000
92 ->100 0.13471
93 ->100 -0.12296
93 ->102 -0.14336
94 ->100 0.14998
95 ->100 -0.37001
95 ->101 -0.17127
95 ->103 0.38185
95 ->104 -0.17428
95 ->105 -0.14274

Excited State 35: Singlet-A 5.2937 eV 234.21 nm f=0.0188 <S**2>=0.000
92 ->100 -0.10391
93 ->100 0.21345
93 ->101 -0.10599
93 ->102 0.14295
94 ->100 -0.11754
94 ->102 -0.11875
95 ->101 -0.11203
95 ->102 0.53471
95 ->103 0.16470

Excited State 36: Singlet-A 5.3431 eV 232.05 nm f=0.0056 <S**2>=0.000
88 -> 99 -0.28011
92 ->100 -0.14045
93 ->100 -0.11764
93 ->101 0.43125

93 ->102 0.13318
94 ->101 -0.24560
95 ->102 0.10904
96 ->103 0.12057
96 ->104 0.15673

Excited State 37: Singlet-A 5.3616 eV 231.25 nm f=0.0335 <S**2>=0.000
88 -> 99 0.19339
92 ->100 0.14626
93 ->100 0.21583
93 ->102 -0.10201
96 ->103 0.21414
96 ->104 0.47227
98 ->107 0.17086

Excited State 38: Singlet-A 5.3863 eV 230.19 nm f=0.0009 <S**2>=0.000
96 ->103 -0.10363
96 ->104 -0.16417
98 ->107 0.53067
98 ->108 -0.31720
98 ->109 0.21995

Excited State 39: Singlet-A 5.4044 eV 229.41 nm f=0.0111 <S**2>=0.000
88 -> 99 0.38281
93 ->101 0.28865
94 ->101 -0.18756
96 ->103 -0.21286
96 ->105 -0.36870

Excited State 40: Singlet-A 5.4104 eV 229.16 nm f=0.0143 <S**2>=0.000
88 -> 99 0.41578
93 ->100 -0.15898
94 ->100 0.10097
96 ->103 0.17611
96 ->105 0.43275

Excited State 41: Singlet-A 5.4329 eV 228.21 nm f=0.1113 <S**2>=0.000
92 ->100 -0.18670
93 ->100 -0.22630
93 ->101 -0.20595
93 ->102 0.10852
94 ->100 0.23906
95 ->104 -0.17345
95 ->105 0.12913
96 ->104 0.23061
96 ->105 -0.20234
97 ->109 -0.10495
98 ->108 -0.23175

Excited State 42: Singlet-A 5.4624 eV 226.98 nm f=0.0629 <S**2>=0.000

93 ->100 -0.12319
98 ->107 0.24225
98 ->108 0.54912
98 ->109 0.24235

Excited State 43: Singlet-A 5.4992 eV 225.46 nm f=0.0067 <S**2>=0.000
91 ->100 0.18544
91 ->101 0.11701
94 ->100 -0.10505
95 ->104 0.21429
97 ->106 0.15261
97 ->107 0.42119
97 ->108 0.11559
97 ->109 -0.25892

Excited State 44: Singlet-A 5.5461 eV 223.55 nm f=0.0495 <S**2>=0.000
93 ->100 -0.14355
93 ->101 -0.11238
95 ->103 0.20120
95 ->104 0.46912
96 ->104 0.16109
96 ->109 0.14794
97 ->106 -0.25564

Excited State 45: Singlet-A 5.5817 eV 222.13 nm f=0.0886 <S**2>=0.000
95 ->103 0.23028
95 ->104 0.11045
95 ->105 0.35609
97 ->106 0.45026
97 ->109 0.15709

Excited State 46: Singlet-A 5.5861 eV 221.95 nm f=0.0181 <S**2>=0.000
94 ->100 -0.11619
95 ->103 0.13520
95 ->104 -0.13173
95 ->105 0.45759
96 ->105 0.11937
97 ->106 -0.38761
97 ->110 -0.12213

Excited State 47: Singlet-A 5.6152 eV 220.80 nm f=0.0082 <S**2>=0.000
95 ->105 0.10156
98 ->106 -0.10331
98 ->109 -0.11123
98 ->110 0.64862

Excited State 48: Singlet-A 5.6302 eV 220.21 nm f=0.0040 <S**2>=0.000
91 ->100 0.13511
93 ->100 0.15186
93 ->102 0.19655

93 ->103 -0.14082
94 ->100 0.19960
94 ->102 0.33133
94 ->103 -0.27731
95 ->104 0.14436
97 ->107 -0.21237
97 ->109 -0.20267

Excited State 49: Singlet-A 5.6473 eV 219.55 nm f=0.0132 <S**2>=0.000
91 ->100 -0.10383
93 ->102 0.25986
94 ->102 0.41443
94 ->103 0.11455
97 ->106 -0.10753
97 ->107 0.33523
97 ->109 0.26843

Excited State 50: Singlet-A 5.6808 eV 218.25 nm f=0.0029 <S**2>=0.000
93 ->100 -0.13002
93 ->102 0.11283
93 ->103 0.12402
94 ->100 -0.15012
94 ->101 -0.11715
94 ->102 0.20598
94 ->103 0.21632
94 ->105 -0.10792
95 ->105 -0.10899
95 ->109 -0.12885
97 ->107 -0.28167
97 ->108 0.33547
97 ->109 -0.11861

Excited State 51: Singlet-A 5.7250 eV 216.57 nm f=0.0022 <S**2>=0.000
91 ->100 0.13920
91 ->101 -0.22501
92 ->100 -0.21913
92 ->101 0.51817
97 ->108 0.17575
98 ->110 -0.12061

Excited State 52: Singlet-A 5.7288 eV 216.42 nm f=0.0064 <S**2>=0.000
92 ->101 -0.12239
94 ->102 -0.13051
94 ->103 -0.15119
95 ->109 0.10911
97 ->108 0.56550
97 ->109 0.16626

Excited State 53: Singlet-A 5.7738 eV 214.73 nm f=0.0353 <S**2>=0.000
91 ->100 -0.12474

92 ->100 0.11135
94 ->103 0.22670
95 ->103 0.14703
95 ->107 -0.18352
95 ->109 0.31759
96 ->107 0.17782
96 ->109 -0.29269
97 ->109 -0.16682

Excited State 54: Singlet-A 5.8251 eV 212.84 nm f=0.0131 <S**2>=0.000
90 ->101 -0.13524
91 ->100 0.32119
92 ->100 -0.15136
92 ->101 -0.15876
93 ->103 0.39122
94 ->104 0.10197
95 ->109 0.10490
97 ->109 0.23140

Excited State 55: Singlet-A 5.8380 eV 212.37 nm f=0.2316 <S**2>=0.000
90 ->101 0.21797
91 ->100 0.29272
92 ->101 -0.15887
92 ->102 0.10940
93 ->100 0.16771
93 ->102 -0.14107
93 ->103 -0.19095
94 ->103 0.21508
95 ->107 -0.10026
95 ->109 0.16055
97 ->110 0.24578

Excited State 56: Singlet-A 5.8624 eV 211.49 nm f=0.0951 <S**2>=0.000
91 ->101 0.56407
92 ->100 -0.12303
92 ->101 0.25523
93 ->103 0.12814

Excited State 57: Singlet-A 5.9017 eV 210.08 nm f=0.0051 <S**2>=0.000
90 ->100 0.44769
91 ->100 0.19937
92 ->100 0.20057
93 ->102 0.12000
94 ->103 0.12077
94 ->104 0.18372
94 ->105 -0.11722

Excited State 58: Singlet-A 5.9112 eV 209.74 nm f=0.0254 <S**2>=0.000
90 ->100 0.18034
90 ->101 0.34187

91 ->100 0.11550
93 ->102 0.12818
93 ->103 0.19375
93 ->104 -0.10692
94 ->103 -0.21651
94 ->104 -0.29975
94 ->105 0.11736
97 ->110 0.13417

Excited State 59: Singlet-A 5.9499 eV 208.38 nm f=0.0452 <S**2>=0.000
89 ->100 -0.27017
89 ->101 -0.13279
89 ->103 -0.10660
90 ->100 0.30715
91 ->102 0.16810
92 ->100 -0.16091
92 ->103 -0.12703
93 ->102 -0.18782
94 ->102 0.10933
96 ->106 0.17551
97 ->109 -0.13089
97 ->110 -0.10375

Excited State 60: Singlet-A 5.9706 eV 207.66 nm f=0.0373 <S**2>=0.000
91 ->102 0.15015
92 ->102 -0.12929
94 ->103 0.14570
94 ->104 0.14727
94 ->105 0.22410
95 ->107 0.12861
95 ->109 -0.17982
96 ->106 0.31543
96 ->109 -0.12645
97 ->110 0.27514

Excited State 61: Singlet-A 5.9856 eV 207.14 nm f=0.0565 <S**2>=0.000
89 ->100 -0.10716
90 ->101 -0.30054
91 ->102 -0.13375
92 ->102 0.15387
93 ->105 -0.11904
94 ->103 -0.15355
94 ->104 -0.23919
94 ->105 -0.22121
96 ->106 0.31202
97 ->110 0.22253

Excited State 62: Singlet-A 5.9956 eV 206.79 nm f=0.0046 <S**2>=0.000
89 ->100 -0.22138
89 ->102 0.10272

91 ->102 -0.22995
92 ->102 0.15386
92 ->103 -0.10482
93 ->102 0.11869
93 ->103 0.25168
93 ->104 0.20938
94 ->103 0.10079
94 ->104 0.16940
94 ->105 0.32452
96 ->106 0.11215

Excited State 63: Singlet-A 6.0344 eV 205.46 nm f=0.0210 <S**2>=0.000
90 ->100 -0.22382
90 ->101 0.19861
91 ->101 0.14283
91 ->102 -0.11559
92 ->103 0.10113
93 ->104 -0.23837
93 ->105 0.15229
94 ->104 0.11127
94 ->105 -0.13371
95 ->106 -0.11298
96 ->106 0.37221
97 ->110 -0.17900

Excited State 64: Singlet-A 6.0433 eV 205.16 nm f=0.0523 <S**2>=0.000
89 ->101 -0.19078
91 ->105 -0.11229
93 ->103 0.11779
93 ->104 -0.11236
93 ->105 0.28969
94 ->104 0.22590
94 ->105 -0.24296
96 ->106 -0.18964
97 ->110 0.28952

Excited State 65: Singlet-A 6.0496 eV 204.95 nm f=0.0146 <S**2>=0.000
89 ->101 0.38400
90 ->101 0.12654
91 ->102 0.16350
93 ->103 0.11343
93 ->104 0.35372
93 ->105 0.21025
94 ->105 -0.23014

Excited State 66: Singlet-A 6.0908 eV 203.56 nm f=0.0426 <S**2>=0.000
89 ->100 -0.14472
89 ->101 0.40914
91 ->100 -0.10885
91 ->102 -0.21765

92 ->103 -0.24456
93 ->104 -0.25940
93 ->105 -0.10213
96 ->106 -0.11995

Excited State 67: Singlet-A 6.1034 eV 203.14 nm f=0.0772 <S**2>=0.000
89 ->100 0.33519
89 ->101 -0.14344
90 ->101 0.19505
91 ->100 -0.10927
91 ->102 -0.14067
92 ->100 -0.11953
92 ->102 -0.10098
92 ->103 -0.32914
93 ->104 0.22213
93 ->105 -0.13598
96 ->106 0.13012

Excited State 68: Singlet-A 6.1292 eV 202.29 nm f=0.0188 <S**2>=0.000
85 -> 99 -0.10550
86 -> 99 -0.10421
89 ->100 0.19524
90 ->100 0.12506
90 ->101 -0.15786
91 ->103 0.12865
92 ->103 -0.15551
93 ->104 -0.10136
93 ->105 0.36869
94 ->104 -0.17330
95 ->106 -0.13414
96 ->107 -0.25322

Excited State 69: Singlet-A 6.1555 eV 201.42 nm f=0.0587 <S**2>=0.000
87 -> 99 -0.12332
93 ->105 0.18174
94 ->104 -0.10314
95 ->106 0.15075
96 ->107 0.51099
96 ->108 -0.20830
96 ->109 0.20652

Excited State 70: Singlet-A 6.1757 eV 200.76 nm f=0.0437 <S**2>=0.000
91 ->103 -0.21897
92 ->102 0.23214
93 ->104 0.10305
93 ->105 0.12988
95 ->106 0.40846
96 ->107 -0.11713
96 ->108 0.27285
97 ->110 -0.11208

Excited State 71: Singlet-A 6.2006 eV 199.96 nm f=0.0938 <S**2>=0.000
90 ->102 0.47471
91 ->102 0.28641
91 ->103 -0.20338
92 ->102 0.18380
92 ->103 -0.10824
95 ->106 -0.15357

Excited State 72: Singlet-A 6.2024 eV 199.90 nm f=0.0563 <S**2>=0.000
91 ->102 -0.11402
91 ->103 -0.12743
92 ->103 0.11190
95 ->106 -0.40753
96 ->107 0.16698
96 ->108 0.40947
96 ->109 0.15292
SavETr: write IOETrn= 770 NScale= 10 NData= 16 NLR=1 NState= 72 LETran= 1306.

Table S4. List of excited states, excitation energy (eV), wavelength (nm), oscillator strength (f) and MOs contributions for compound **2** at b3lyp/sdd with PCM in chloroform.

Excited State 1: Singlet-A 2.5464 eV 486.91 nm f=0.0030 <S**2>=0.000
111 ->112 0.69751
This state for optimization and/or second-order correction.
Total Energy, E(TD-HF/TD-KS) = -1286.09485888
Copying the excited state density for this state as the 1-particle RhoCI density.

Excited State 2: Singlet-A 3.0231 eV 410.13 nm f=0.0662 <S**2>=0.000
107 ->112 0.10089
110 ->112 0.61730
111 ->114 0.22650
111 ->118 0.13861
111 ->119 -0.12229

Excited State 3: Singlet-A 3.2663 eV 379.58 nm f=0.0248 <S**2>=0.000
110 ->112 -0.29595
111 ->114 0.46789
111 ->116 0.14650
111 ->117 0.11025
111 ->118 0.25490
111 ->119 -0.21844

Excited State 4: Singlet-A 3.3698 eV 367.92 nm f=0.0314 <S**2>=0.000
108 ->112 0.50642
109 ->112 -0.45078

Excited State 5: Singlet-A 3.4021 eV 364.43 nm f=0.0117 <S**2>=0.000
111 ->113 0.50806
111 ->115 -0.34875

111 ->116 0.14158
111 ->117 0.20540
111 ->118 -0.11899

Excited State 6: Singlet-A 3.5622 eV 348.06 nm f=0.2166 <S**2>=0.000
108 ->112 0.44827
109 ->112 0.50937
110 ->113 0.10538

Excited State 7: Singlet-A 3.6406 eV 340.56 nm f=0.0021 <S**2>=0.000
108 ->114 0.43535
108 ->116 0.11949
108 ->118 0.29074
108 ->119 -0.25291
109 ->114 -0.22202
109 ->118 -0.14031
109 ->119 0.12187

Excited State 8: Singlet-A 3.8702 eV 320.35 nm f=0.0522 <S**2>=0.000
106 ->112 0.13310
107 ->112 0.57068
109 ->113 0.10140
110 ->114 -0.23994
110 ->116 -0.10011
110 ->118 -0.10017

Excited State 9: Singlet-A 3.8787 eV 319.65 nm f=0.0249 <S**2>=0.000
107 ->112 0.29286
107 ->114 0.14875
110 ->113 0.17343
110 ->114 0.44517
110 ->118 0.19374
110 ->119 -0.15555

Excited State 10: Singlet-A 3.9924 eV 310.55 nm f=0.2079 <S**2>=0.000
105 ->112 0.10471
108 ->113 0.15126
108 ->115 -0.11862
109 ->114 0.18565
109 ->118 0.11672
109 ->119 -0.10189
110 ->113 0.51319
110 ->114 -0.13796

Excited State 11: Singlet-A 4.0065 eV 309.46 nm f=0.0879 <S**2>=0.000
109 ->114 0.14629
110 ->113 -0.28663
111 ->113 0.38753
111 ->115 0.37129
111 ->117 -0.10695

Excited State 12: Singlet-A 4.0293 eV 307.71 nm f=0.0109 <S**2>=0.000
105 ->112 0.55403
106 ->112 -0.18362
109 ->114 -0.17836
109 ->118 -0.11278

Excited State 13: Singlet-A 4.0432 eV 306.65 nm f=0.0805 <S**2>=0.000
105 ->112 -0.27498
106 ->112 0.18253
108 ->113 -0.16514
108 ->115 0.11895
109 ->114 -0.17530
109 ->118 -0.11387
110 ->113 0.27296
111 ->113 0.21718
111 ->115 0.29738

Excited State 14: Singlet-A 4.0873 eV 303.34 nm f=0.0108 <S**2>=0.000
105 ->112 0.24647
106 ->112 0.58485
107 ->112 -0.17272
110 ->117 0.15055

Excited State 15: Singlet-A 4.2396 eV 292.44 nm f=0.0076 <S**2>=0.000
111 ->114 -0.32492
111 ->115 0.12372
111 ->116 0.55454
111 ->118 0.18140

Excited State 16: Singlet-A 4.2899 eV 289.02 nm f=0.0543 <S**2>=0.000
108 ->113 -0.17413
108 ->115 0.10747
109 ->113 0.43102
109 ->114 0.17383
109 ->115 -0.20302
109 ->116 0.14368
109 ->117 0.17431
111 ->115 -0.15358
111 ->116 -0.19732

Excited State 17: Singlet-A 4.3495 eV 285.06 nm f=0.0700 <S**2>=0.000
109 ->113 -0.10466
110 ->115 0.36064
110 ->116 -0.17119
111 ->114 -0.23971
111 ->115 -0.14063
111 ->116 -0.22041
111 ->118 0.33284
111 ->119 -0.22265

Excited State 18: Singlet-A 4.3666 eV 283.94 nm f=0.0631 <S**2>=0.000
104 ->112 -0.22644
108 ->113 -0.11161
110 ->115 0.43878
110 ->116 -0.16747
111 ->114 0.16634
111 ->116 0.15478
111 ->118 -0.24957
111 ->119 0.13059

Excited State 19: Singlet-A 4.3951 eV 282.10 nm f=0.0085 <S**2>=0.000
103 ->112 -0.25487
104 ->112 0.56372
105 ->112 -0.10027
109 ->113 0.10283
110 ->115 0.15692
111 ->118 -0.10700

Excited State 20: Singlet-A 4.4594 eV 278.03 nm f=0.0450 <S**2>=0.000
103 ->112 -0.10256
104 ->112 -0.13362
108 ->113 0.39171
108 ->115 -0.19733
108 ->117 0.10671
109 ->113 0.27430
109 ->114 -0.16838
111 ->115 0.10390
111 ->117 0.13752

Excited State 21: Singlet-A 4.4814 eV 276.66 nm f=0.0017 <S**2>=0.000
104 ->112 0.14207
110 ->114 -0.10643
110 ->116 0.13871
111 ->115 0.22126
111 ->117 0.57593
111 ->118 -0.10065

Excited State 22: Singlet-A 4.4869 eV 276.32 nm f=0.0229 <S**2>=0.000
110 ->114 -0.26757
110 ->115 0.26812
110 ->116 0.51646
110 ->118 0.10556
111 ->117 -0.17406

Excited State 23: Singlet-A 4.5292 eV 273.74 nm f=0.0023 <S**2>=0.000
102 ->112 0.14404
103 ->112 0.58050
104 ->112 0.26920
108 ->113 0.10585

Excited State 24: Singlet-A 4.5752 eV 270.99 nm f=0.0209 <S**2>=0.000
107 ->114 -0.13484
110 ->114 0.27908
110 ->116 0.26338
110 ->118 -0.26923
110 ->119 0.18828
111 ->118 0.18269
111 ->119 0.26042
111 ->123 -0.20024

Excited State 25: Singlet-A 4.6138 eV 268.73 nm f=0.0050 <S**2>=0.000
110 ->114 -0.17324
110 ->116 -0.15210
110 ->117 0.10269
110 ->118 0.23506
110 ->119 -0.13178
111 ->118 0.28145
111 ->119 0.43367
111 ->123 -0.17399

Excited State 26: Singlet-A 4.7172 eV 262.83 nm f=0.0284 <S**2>=0.000
106 ->113 -0.12829
107 ->113 0.13100
107 ->115 -0.10147
109 ->113 0.28721
109 ->115 0.33937
109 ->116 -0.15350
109 ->117 -0.12957
110 ->117 0.33742
110 ->118 -0.15251
111 ->123 0.10176

Excited State 27: Singlet-A 4.7776 eV 259.51 nm f=0.0009 <S**2>=0.000
102 ->112 0.18113
110 ->117 -0.10417
110 ->118 -0.14902
111 ->118 0.17144
111 ->119 0.25806
111 ->120 -0.23905
111 ->121 0.16748
111 ->122 0.19099
111 ->123 0.39910

Excited State 28: Singlet-A 4.7972 eV 258.45 nm f=0.0035 <S**2>=0.000
102 ->112 0.63838
103 ->112 -0.19430
111 ->123 -0.11113

Excited State 29: Singlet-A 4.8574 eV 255.25 nm f=0.1077 <S**2>=0.000

106 ->113 -0.11223
107 ->113 0.11854
108 ->113 0.20692
108 ->115 0.22826
109 ->113 -0.19921
109 ->114 0.12245
109 ->115 -0.33684
110 ->117 0.34780
110 ->118 -0.13864

Excited State 30: Singlet-A 4.8836 eV 253.88 nm f=0.0039 <S**2>=0.000
107 ->113 -0.13089
107 ->114 -0.22540
107 ->119 0.10230
108 ->113 0.11320
110 ->118 0.43334
110 ->119 0.30611
111 ->123 0.12851

Excited State 31: Singlet-A 4.9207 eV 251.96 nm f=0.0091 <S**2>=0.000
105 ->113 0.10114
106 ->113 0.23443
107 ->113 0.52624
109 ->117 0.11587
110 ->118 0.15101
110 ->119 0.20219

Excited State 32: Singlet-A 4.9567 eV 250.14 nm f=0.0498 <S**2>=0.000
105 ->114 0.10666
106 ->114 0.12131
107 ->113 -0.11209
107 ->114 0.31963
107 ->118 0.12920
108 ->113 0.12273
108 ->115 0.12615
109 ->114 0.15062
110 ->117 -0.17996
110 ->119 0.40136

Excited State 33: Singlet-A 4.9704 eV 249.45 nm f=0.1012 <S**2>=0.000
107 ->114 -0.11578
107 ->115 -0.11385
108 ->113 0.31182
108 ->115 0.37748
108 ->117 -0.10382
109 ->115 0.14538
110 ->117 -0.21720
110 ->119 -0.26773

Excited State 34: Singlet-A 4.9908 eV 248.43 nm f=0.0127 <S**2>=0.000

108 ->115 0.10233
108 ->116 -0.12560
109 ->114 -0.25081
109 ->115 0.15548
109 ->116 0.53474
109 ->118 0.14439
110 ->117 0.10780

Excited State 35: Singlet-A 5.0625 eV 244.91 nm f=0.1642 <S**2>=0.000
101 ->112 0.24761
105 ->113 0.18055
106 ->113 0.22997
107 ->113 -0.19935
107 ->115 0.15871
107 ->116 -0.10452
108 ->115 0.12946
109 ->114 0.15238
109 ->115 0.23971
109 ->117 0.17012
109 ->118 -0.18902
109 ->119 0.10943
110 ->117 0.19143

Excited State 36: Singlet-A 5.0849 eV 243.83 nm f=0.0021 <S**2>=0.000
104 ->114 0.16642
105 ->113 0.34807
105 ->116 0.13683
108 ->115 0.12153
108 ->116 0.18644
109 ->114 -0.23993
109 ->116 -0.16736
109 ->118 0.28509
109 ->119 -0.17398

Excited State 37: Singlet-A 5.1142 eV 242.43 nm f=0.0373 <S**2>=0.000
104 ->114 0.11955
105 ->113 0.24357
105 ->114 -0.12031
105 ->116 0.11841
106 ->113 -0.27731
107 ->113 0.12816
108 ->114 -0.12552
108 ->115 -0.10007
108 ->116 0.16911
109 ->114 0.13864
109 ->116 0.30259
109 ->117 -0.10310
109 ->118 -0.23430
109 ->119 0.12885

Excited State 38: Singlet-A 5.1709 eV 239.77 nm f=0.0042 <S**2>=0.000
105 ->113 -0.36502
108 ->114 -0.26648
108 ->115 0.13091
108 ->116 0.45531
108 ->118 0.11746

Excited State 39: Singlet-A 5.2199 eV 237.52 nm f=0.0643 <S**2>=0.000
101 ->112 0.57526
106 ->113 -0.14597
107 ->115 -0.14421
107 ->116 0.12043
109 ->115 -0.11405
109 ->117 -0.18759

Excited State 40: Singlet-A 5.2461 eV 236.34 nm f=0.0086 <S**2>=0.000
108 ->114 0.15852
108 ->118 -0.14253
111 ->120 0.40029
111 ->121 0.46022

Excited State 41: Singlet-A 5.2513 eV 236.10 nm f=0.0038 <S**2>=0.000
104 ->114 0.14005
105 ->113 -0.16724
105 ->114 -0.13369
105 ->115 0.13042
107 ->115 -0.15237
108 ->114 0.26711
108 ->117 -0.18330
108 ->118 -0.23140
108 ->119 0.14995
109 ->117 0.18878
111 ->120 -0.19706
111 ->121 -0.22795

Excited State 42: Singlet-A 5.2670 eV 235.40 nm f=0.0199 <S**2>=0.000
104 ->114 -0.10085
105 ->114 0.13672
106 ->113 -0.25174
107 ->115 -0.12679
107 ->116 0.11438
108 ->115 -0.10854
108 ->117 -0.20574
108 ->118 0.14582
109 ->115 0.10547
109 ->117 0.43190

Excited State 43: Singlet-A 5.2911 eV 234.33 nm f=0.0120 <S**2>=0.000
100 ->112 0.10310
104 ->114 -0.19652

105 ->113 0.11919
105 ->114 0.11728
105 ->115 -0.19727
105 ->116 -0.13777
106 ->114 -0.11391
108 ->114 0.17532
108 ->115 0.17911
108 ->116 0.34217
108 ->118 -0.21712
108 ->119 0.18291

Excited State 44: Singlet-A 5.3247 eV 232.85 nm f=0.1513 <S**2>=0.000
105 ->115 0.11130
106 ->113 -0.19439
106 ->115 0.11009
106 ->116 -0.10509
106 ->117 -0.13014
107 ->113 0.21731
107 ->115 0.42177
107 ->117 -0.14465
109 ->118 0.11672
109 ->119 0.13560

Excited State 45: Singlet-A 5.3424 eV 232.08 nm f=0.0023 <S**2>=0.000
100 ->112 0.58349
103 ->112 -0.10417
105 ->113 -0.11337
107 ->115 -0.13353
108 ->117 0.13552
109 ->117 0.16911

Excited State 46: Singlet-A 5.3603 eV 231.30 nm f=0.0287 <S**2>=0.000
104 ->114 -0.10019
105 ->114 -0.18119
109 ->118 0.34550
109 ->119 0.44018
111 ->120 -0.14102
111 ->121 0.14658

Excited State 47: Singlet-A 5.3866 eV 230.17 nm f=0.0091 <S**2>=0.000
107 ->115 -0.15126
109 ->118 0.13122
109 ->119 0.19595
111 ->120 0.38030
111 ->121 -0.35262
111 ->122 0.26645
111 ->123 0.22183

Excited State 48: Singlet-A 5.4021 eV 229.51 nm f=0.1199 <S**2>=0.000
100 ->112 -0.14657

104 ->114 0.10496
105 ->114 0.27770
106 ->113 -0.11634
107 ->114 -0.16051
107 ->115 -0.11781
108 ->115 0.13624
108 ->117 0.39411
108 ->118 -0.13811
109 ->119 0.16875
111 ->120 -0.12286

Excited State 49: Singlet-A 5.4220 eV 228.67 nm f=0.0033 <S**2>=0.000
100 ->112 -0.17269
104 ->113 0.12043
105 ->114 -0.18534
105 ->115 -0.10131
106 ->114 -0.13807
107 ->114 0.32693
107 ->116 -0.20250
107 ->118 -0.10568
108 ->117 0.28539
109 ->119 -0.11789

Excited State 50: Singlet-A 5.4484 eV 227.56 nm f=0.0507 <S**2>=0.000
104 ->113 0.15856
105 ->114 -0.26954
106 ->114 0.29066
107 ->114 -0.13129
107 ->116 0.28903
108 ->117 0.19339
109 ->117 0.10532
110 ->120 0.14370
111 ->122 0.10437

Excited State 51: Singlet-A 5.4757 eV 226.42 nm f=0.0053 <S**2>=0.000
104 ->113 -0.36130
105 ->114 -0.14068
106 ->114 -0.26417
107 ->115 0.16067
107 ->116 0.40526

Excited State 52: Singlet-A 5.4774 eV 226.35 nm f=0.0244 <S**2>=0.000
103 ->114 0.20512
104 ->113 -0.17140
107 ->114 0.11799
107 ->123 -0.10487
110 ->120 0.36602
110 ->121 -0.20737
110 ->122 -0.17097
110 ->123 -0.26170

Excited State 53: Singlet-A 5.4910 eV 225.80 nm f=0.0120 <S**2>=0.000
104 ->113 0.41025
104 ->114 0.10893
105 ->116 0.14255
106 ->114 -0.21024
107 ->116 0.22606
111 ->122 -0.31411
111 ->123 0.17332

Excited State 54: Singlet-A 5.4981 eV 225.50 nm f=0.0469 <S**2>=0.000
104 ->113 0.19706
105 ->116 0.11899
106 ->114 -0.19442
111 ->120 -0.11791
111 ->122 0.47178
111 ->123 -0.30058

Excited State 55: Singlet-A 5.5481 eV 223.47 nm f=0.0061 <S**2>=0.000
106 ->114 0.18154
108 ->118 0.30820
108 ->119 0.47907
108 ->123 -0.17747
109 ->119 0.13968
109 ->123 0.10714

Excited State 56: Singlet-A 5.5632 eV 222.87 nm f=0.0014 <S**2>=0.000
104 ->113 0.15875
105 ->113 0.11432
105 ->115 0.43747
105 ->116 -0.34537
105 ->117 -0.10611
106 ->115 -0.21379
106 ->116 0.15266
108 ->119 0.10455

Excited State 57: Singlet-A 5.5948 eV 221.61 nm f=0.0342 <S**2>=0.000
105 ->114 -0.10624
105 ->115 0.11343
105 ->116 -0.10252
106 ->114 -0.12224
106 ->115 0.12030
106 ->116 -0.13021
107 ->114 -0.13372
107 ->117 0.26758
107 ->118 0.28165
107 ->119 -0.11271
108 ->118 0.10720
110 ->120 0.28373
110 ->121 0.21069

110 ->123 0.15539

Excited State 58: Singlet-A 5.6085 eV 221.06 nm f=0.0265 <S**2>=0.000
105 ->114 0.16568
106 ->114 0.14351
107 ->116 0.12349
107 ->118 -0.28557
107 ->119 0.17354
108 ->118 -0.12855
108 ->119 -0.10663
110 ->120 0.24982
110 ->121 0.37211

Excited State 59: Singlet-A 5.6238 eV 220.46 nm f=0.0083 <S**2>=0.000
103 ->113 0.10742
105 ->115 0.10280
106 ->115 0.33385
106 ->116 -0.16923
107 ->117 0.27292
107 ->118 -0.11523
110 ->120 -0.31843
110 ->123 -0.21654

Excited State 60: Singlet-A 5.6461 eV 219.59 nm f=0.0257 <S**2>=0.000
102 ->113 0.10335
103 ->113 0.60339
104 ->113 0.13189
107 ->117 -0.12220

Excited State 61: Singlet-A 5.6597 eV 219.07 nm f=0.0082 <S**2>=0.000
103 ->114 0.16816
106 ->115 -0.13177
107 ->117 -0.14283
107 ->118 0.10733
107 ->119 -0.13810
110 ->120 -0.13685
110 ->121 0.44180
110 ->122 -0.12474
110 ->123 -0.32430

Excited State 62: Singlet-A 5.6951 eV 217.70 nm f=0.1105 <S**2>=0.000
99 ->112 -0.13566
103 ->113 -0.11513
105 ->115 0.21804
105 ->116 0.13850
106 ->113 0.13167
106 ->115 0.41474
106 ->116 0.25780
107 ->115 -0.12189
107 ->117 -0.23882

Excited State 63: Singlet-A 5.7319 eV 216.31 nm f=0.0098 <S**2>=0.000
103 ->114 0.11005
106 ->116 -0.12967
107 ->119 -0.14114
108 ->123 0.15996
110 ->122 0.56144
110 ->123 -0.14070

Excited State 64: Singlet-A 5.7384 eV 216.06 nm f=0.0507 <S**2>=0.000
103 ->114 0.16026
106 ->116 -0.21644
107 ->118 -0.12810
107 ->119 0.12461
108 ->118 0.11668
108 ->119 0.13893
108 ->120 -0.16208
108 ->121 0.12120
108 ->122 0.13591
108 ->123 0.27406
109 ->120 0.10892
109 ->123 -0.15245
110 ->122 -0.24746
110 ->123 0.26045

Excited State 65: Singlet-A 5.7684 eV 214.94 nm f=0.2176 <S**2>=0.000
99 ->112 0.21703
103 ->114 -0.14952
105 ->116 0.11791
106 ->116 0.37334
107 ->117 0.23166
107 ->118 -0.17489
108 ->119 0.11587
108 ->120 -0.14032
108 ->122 0.10474
108 ->123 0.23161

Excited State 66: Singlet-A 5.8415 eV 212.25 nm f=0.1018 <S**2>=0.000
99 ->112 -0.15214
103 ->114 -0.24848
103 ->115 0.10477
104 ->114 0.26160
104 ->115 -0.13024
105 ->116 -0.24024
105 ->118 -0.24305
106 ->117 0.22881
106 ->118 0.10804
108 ->123 0.10716
110 ->123 -0.13194

Excited State 67: Singlet-A 5.8545 eV 211.78 nm f=0.0237 <S**2>=0.000
99 ->112 0.11186
102 ->113 -0.34098
106 ->117 -0.17756
107 ->118 0.27127
107 ->119 0.39669
110 ->122 0.12561

Excited State 68: Singlet-A 5.8640 eV 211.43 nm f=0.0228 <S**2>=0.000
102 ->113 0.51096
103 ->113 -0.11321
104 ->115 0.10964
106 ->117 -0.10673
107 ->118 0.19886
107 ->119 0.25922

Excited State 69: Singlet-A 5.8787 eV 210.90 nm f=0.0371 <S**2>=0.000
102 ->113 -0.11441
103 ->114 -0.16700
103 ->115 -0.15249
104 ->114 -0.14187
104 ->115 0.42660
105 ->114 -0.10292
105 ->117 0.16484
105 ->118 0.16339
106 ->117 0.13118
107 ->119 0.14702
109 ->120 -0.10727
110 ->123 -0.10615

Excited State 70: Singlet-A 5.8970 eV 210.25 nm f=0.0439 <S**2>=0.000
99 ->112 -0.23287
103 ->114 0.14095
105 ->117 -0.23049
106 ->117 0.40977
107 ->117 0.19488
107 ->119 0.16249

Excited State 71: Singlet-A 5.9073 eV 209.88 nm f=0.0120 <S**2>=0.000
102 ->114 -0.15046
104 ->115 -0.10895
104 ->116 0.29413
104 ->118 0.10356
105 ->114 -0.13663
105 ->117 0.18176
105 ->118 0.23028
111 ->124 0.37370

Excited State 72: Singlet-A 5.9202 eV 209.43 nm f=0.0072 <S**2>=0.000
102 ->113 -0.13663

```
   104 ->115         0.21952
   104 ->116        -0.25348
   105 ->117        -0.15646
   105 ->118        -0.11457
   111 ->124         0.51040
SavETr:  write IOETrn=   770 NScale= 10 NData=  16 NLR=1 NState=   72 LETran=    1306.
```
